# A validated mathematical model of FGFR3-mediated tumor growth reveals pathways to harness the benefits of combination targeted therapy and immunotherapy in bladder cancer

**DOI:** 10.1002/cso2.1019

**Published:** 2021-05-19

**Authors:** Kamaldeen Okuneye, Daniel Bergman, Jeffrey C. Bloodworth, Alexander T. Pearson, Randy F. Sweis, Trachette L. Jackson

**Affiliations:** 1 Applied BioMath, LLC, Concord, Massachusetts, USA; 2 Department of Mathematics, University of Michigan, Ann Arbor, Michigan, USA; 3 Section of Hematology/Oncology, Department of Medicine, The University of Chicago, Chicago, Illinois, USA

**Keywords:** bladder cancer, FGFR3-targeted therapy, immune checkpoint inhibitors, mathematical model

## Abstract

Bladder cancer is a common malignancy with over 80,000 estimated new cases and nearly 18,000 deaths per year in the United States alone. Therapeutic options for metastatic bladder cancer had not evolved much for nearly four decades, until recently, when five immune checkpoint inhibitors were approved by the U.S. Food and Drug Administration (FDA). Despite the activity of these drugs in some patients, the objective response rate for each is less than 25%. At the same time, fibroblast growth factor receptors (FGFRs) have been attractive drug targets for a variety of cancers, and in 2019 the FDA approved the first therapy targeted against FGFR3 for bladder cancer. Given the excitement around these new receptor tyrosine kinase and immune checkpoint targeted strategies, and the challenges they each may face on their own, emerging data suggest that combining these treatment options could lead to improved therapeutic outcomes. In this paper, we develop a mathematical model for FGFR3-mediated tumor growth and use it to investigate the impact of the combined administration of a small molecule inhibitor of FGFR3 and a monoclonal antibody against the PD-1/PD-L1 immune checkpoint. The model is carefully calibrated and validated with experimental data before survival benefits, and dosing schedules are explored. Predictions of the model suggest that FGFR3 mutation reduces the effectiveness of anti-PD-L1 therapy, that there are regions of parameter space where each monotherapy can outperform the other, and that pretreatment with anti-PD-L1 therapy always results in greater tumor reduction even when anti-FGFR3 therapy is the more effective monotherapy.

## 1 | INTRODUCTION

Bladder cancer is one of the 10 most common cancers in the United States. The lethality of this disease is driven by Stage II or higher cancers and in these advanced stages, 5-year survival rates are low (below 35%) [1]. For more than 30 years, therapeutic strategies for bladder cancer in Stages II–IV have focused on the use of systemic chemotherapy before, during, or after loco-regional therapy [2]. Unfortunately, outcomes with chemotherapy are poor in advanced cases [3]. For this reason, researchers have turned their attention to targeted therapies.

Members of the fibroblast growth factor receptor (FGFR) family have become a successful therapeutic focal point for bladder cancer [4]. Genomic analysis of bladder cancer has identified frequent alterations of FGFRs, including overexpression and mutations of FGFR3 that activate the receptor via ligand-independent dimerization [4]. Under normal conditions, heparin-bound fibroblast growth factor (FGF) mediates FGFR3 dimerization, leading to kinase activation and stimulation of the extracellular-signal-regulated kinase (ERK) and protein kinase B (AKT) signaling pathways, followed by increased cell proliferation and cell survival [4]. FGFR3 mutations that lead to constitutive activation of downstream signaling pathways in the absence of FGF are commonly found in bladder cancers. Urothelial bladder carcinoma has the most established association with altered FGFR3 signaling, with up to 80% of low-grade tumors harboring FGFR3 mutations [5]. Clinical trials using small molecule inhibitors (SMIs) of FGFR3 show promising clinical responses for patients with FGFR3 mutations, and in 2019, the U.S. Food and Drug Administration (FDA) approved the first therapy targeted against FGFR3 [4].

**FIGURE 1:**
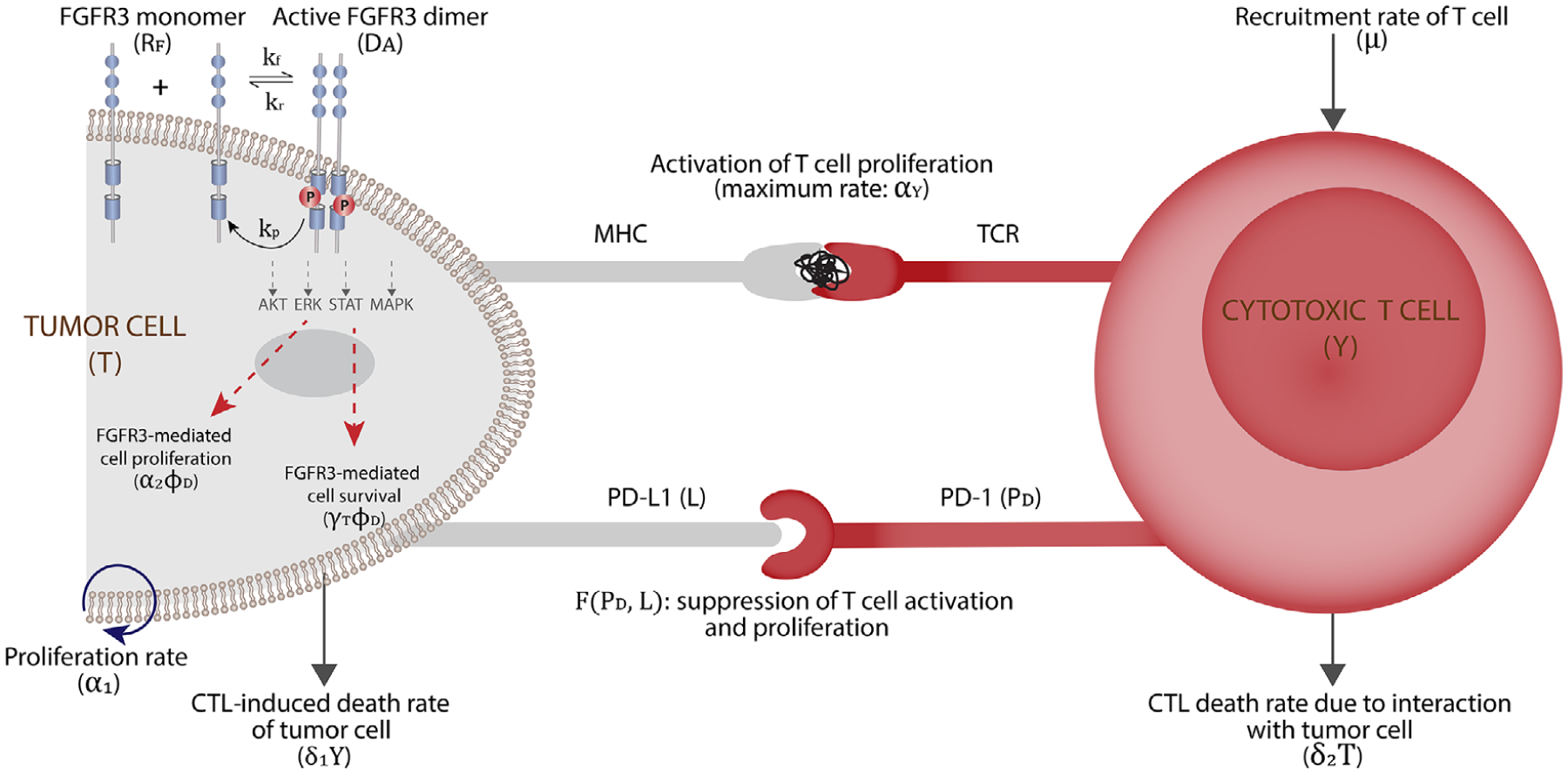
The microenvironment of a tumor cell showing the dynamics of FGFR3 mutation on tumor cells (phosphorylation of the kinase region leads to activation of AKT, ERK, STAT, and MAPK proteins, which result in target DNA transcription leading to cell proliferation and cell survival), the activation of T cell by tumor cells, and suppression of T cell activation and proliferation by PD-L1 binding with PD-1

At the same time, immunotherapy has now emerged as an exciting domain for exploration for many cancers including bladder cancer. The recent success of programmed cell death protein 1 (PD-1) and programmed death-ligand 1 (PD-L1) blockade in cancer therapy illustrates the important role of the PD-1/PD-L1 checkpoint in the regulation of antitumor immune responses [6]. In particular, monoclonal antibodies (mAbs) targeting the PD-1/PD-L1 pathway have resulted in favorable outcomes in advanced bladder cancer and six immune checkpoint inhibitors (ICIs) targeting this pathway were approved in 2015–2018 [7]. Despite the therapeutic potential of ICIs, only a minority (approximately 20%) of bladder cancer patients respond favorably to these therapies and median survival with second-line immunotherapy remains shorter than 1 year [8]. Figure 1 is a schematic diagram showing the impact of FGFR3 mutations and PD-1-PD-L1 checkpoints on tumor growth and tumor cell–T cell interactions.

Given the potential and challenges ICIs on their own, it is possible that the coacting combination of potent ICIs and specific FGFR3 inhibitors can offer much-needed improvements in targeted therapeutics for bladder cancer. The rationale for combining FGFR3-targeted therapy with immunotherapy is confirmed in preclinical and correlative literature and animal models suggest potential synergies between these two mechanisms [8]. When attempting to combine two very different therapeutic approaches that target distinct pathways, treatment outcomes can depend on the order and timing in which therapies are administered. Experimental studies of the most appropriate strategy for FGFR3 inhibition in the context of ICI therapy (either through sequencing or combination) are generally in early clinical stages. Data-driven mathematical modeling is an ideal tool for analyzing novel drug combinations for clinical cancer treatment. For example, multiscale mathematical models have been used to investigate the impact of combined therapies on myeloma cell growth, to predict the effect of receptor tyrosine inhibitor therapy in brain cancer, and to optimize prostate cancer treatment using less drugs [9–11]. Previously published models from our team connect the molecular events associated with tumor growth to the temporal changes in proliferation, migration, and survival of multiple cell types and link these dynamics to tumor growth rates, vascular composition, and therapeutic outcome [12–15]. Evidence of the impact of computational modeling in cancer therapeutics is the fact that our multiscale mathematical model of the VEGF-CXCL8-BCL-2 pathway suggested that metronomic dosing of a SMI of BCl-2 could provide optimal efficacy [13]. These model-based predictions were then validated in a series of preclinical studies [16] and led to the application in a clinical study [14]. In this paper, we design what is to our knowledge, the first model to investigate FGFR3-mediated, advanced bladder tumor growth and response to combination targeted and ICI therapy. We follow our validated approach described above [4–7] to derive a multiscale system of ordinary differential equations that capture the impact of FGFR3 on tumor cell proliferation and survival. To this model, we add the effects of immune surveillance, tumor-immune cell kill, and immune suppression by the growing tumor. Our model of tumor immune dynamics follows closely previously published models [10,12,13,16] of the PD-1/PD-L1 immune checkpoint. The novelty of the work presented here is the combination of both immune checkpoint and receptor tyrosine kinase-mediated tumor growth and therapy. The sections below describe the details of model development, sensitivity and identifiability analysis, and parameter estimation. Most importantly, we use the model to make testable therapeutic predictions at a time when there have been no published experimental studies with combination anti-FGFR therapy and anti-PD-L1 therapy, though preclinical investigations are ongoing.

## 2 | MODEL FORMULATION AND EXPERIMENTAL DATA

Our mathematical model is based on the current biological understanding of bladder cancer growth when the FGFR3 mutation is present. We first develop a pretreatment model that describes the impact of ligand-independent activation of FGFR3 on tumor growth and cytotoxic T cell (CTL) mediated death. This model is based on the validated approach in [12, 15, 17]. Next, we extend the pretreatment model to include anti-PD-L1 therapy alone and in combination with FGFR3-targeted therapy. These models are used to predict the impact of therapy on survival outcomes and to suggest the best dose-scheduling regimes for therapeutic efficacy.

### 2.1 | Model formulation with FGFR3 and immune checkpoints

The pretreatment model, described in detail below, captures the local evolution of free FGFR3 (*R*) and active FGFR3 dimer complexes (*D*) on tumor cells (*T*) as well as PD-1 (*P*_*D*_) and PD-L1 (*L*) mediated immune cell (*Y*) kill. The model variables and their units are described in Table 1.

The equations in (1) describe the ligand-independent dimerization of FGFR3. Parameters that mediate these FGFR3 dynamics include the receptor association rate (*k*_*f*_) and dissociation rate (*k*_*r*_). It is also known that activated receptors undergo stimulated endocytosis but can continue to signal along the endocytic pathway [15] so we also include terms for receptor internalization and recycling rate (*k*_*p*_). For a full list of parameters, see Table 2.

(1) $$\begin{array}{l} \frac{dR}{dt}=-2k_{f}R^{2}+2k_{r}D+2k_{p}D+R_{T}P\left(T,\phi_{D}\right)-\frac{R}{R+2D}R_{T}D\left(T,Y,\phi_{D}\right)\\ \frac{dD}{dt}=k_{f}R^{2}-k_{r}D-k_{p}D-\frac{D}{R+2D}R_{T}D\left(T,Y,\phi_{D}\right) \end{array},$$

These oridinary differential equations (ODEs) must account for changes in receptor number due to cellular proliferation and apoptosis. Following [12, 15, 17], the last two terms in the equation for free receptors (*R*) describes the generation of new receptors as cells divide and the loss of receptors as cells die, respectively, where *R*_*T*_ is the total number of FGFR3 molecules on tumor cells and $\frac{R}{R+2D}$ is the fraction of free FGFR3 that is removed from the loss of tumor cells by cytotoxic T cells (*Y*). The FGFR-dependent proliferation growth and death rates of tumor cells (i.e., $P\left(T,\phi_{D}\right)$ and $D\left(T,Y,\phi_{D}\right)$ are defined in the temporal dynamics of the tumor cells described in Equation (3), where *ϕ*_*D*_ is the fractional occupancy of active FGFR3 dimer per cell defined by

(2) $$\phi_{D}=\frac{1}{R_{T}}\frac{D}{T}.$$

Equation (3) models the temporal dynamics of the tumor cells

(3) $$\frac{dT}{dt}=\left(\alpha_{1}+\alpha_{2}\phi_{D}\right)T-\frac{\delta_{1}Y}{1+\gamma_{T}\phi_{D}}T\equiv P\left(T,\phi_{D}\right)-D\left(T,Y,\phi_{D}\right).$$

The first term in Equation (3) describes tumor cells proliferating exponentially with a natural growth rate *α*_1_, and an FGFR-mediated tumor growth rate *α*_2_. The second term in Equation (3) describes the killing of tumor cells by cytotoxic T cells (*Y*) modified by the impact of FGFR3 on tumor survival, where *δ*_1_ is the death rate of a tumor cell by cytotoxic T cells, and *γ*_*T*_ is the sensitivity of fractional occupancy of FGFR. Equation (3) allows us to simulate tumor growth in the absence of the FGFR3 mutation by setting *α*_2_ = *γ*_*T*_ = 0. This formulation assumes that the total number (converted to nmol using molecular weight) of receptors per tumor cell *R*_*T*_ remains constant. This means that the total amount of FGFR3 in the system should be conserved. We can ensure that the model equations do conserve FGFR3 by considering the sum of the equations of the model (1):

$$\frac{dR}{dt}+2\frac{dD}{dt}=R_{T}\left[P\left(T,\phi_{D}\right)-D\left(T,Y,\phi_{D}\right)\right]=R_{T}\frac{dT}{dt}.$$

Therefore, upon integration, we have

$$R+2D=R_{T}T.$$

The equation for the change in cytotoxic T cells (*Y*) is given by:

(4) $$\frac{dY}{dt}=\left(\mu +\alpha_{Y}\frac{T}{\kappa +T}Y\right)F\left(P_{D},L\right)-\delta_{2}TY-\delta_{Y}Y.$$

The first term in Equation (4) represents a constant recruitment/activation of T cells at a rate, *μ*. The second term describes proliferation that occurs as the result of antigenic stimulation by the tumor cells. The maximum proliferation rate is *α*_*Y*_, and *κ* represents the population of *T* at which the immune cells lyse tumor cells at half of their maximum killing rate [18]. The factor *F*(*P*_*D*_, *L*), which is described in greater detail below, represents the suppression of T cell activation and proliferation via the PD-1/PD-L1 checkpoint. The variables *P*_*D*_ and *L* denote the molar concentrations of PD-1 and PD-L1, respectively, expressed by cells within the model. The molar concentrations are obtained by first calculating the PD-1 expression on all T cells and the PD-L1 expression on all T cells and tumor cells as outlined in the Appendix found in [18]. Our formulation of *F*(*P*_*D*_, *L*) in Equation (7) ensures that as *P*_*D*_ and *L* increases so does the number of PD-1/PD-L1 complexes within the tumor region. This increase corresponds to a smaller *F*(*P*_*D*_, *L*) value, modeling the inhibition of T cell activity. Finally, the last two terms describe how CTLs can die. Specifically, interaction with tumor cells can result in death at a rate *δ*_2_ as was done in [19], but which sets our model apart from [18, 20, 21]. CTLs can also die naturally at a rate *δ*_*Y*_.

We assume that all T cells express PD-1 and that the temporal dynamics of this cell-bound protein is proportional to the rate of change of the T cells on which they reside as described by Equation (5). This is the same approach used in [18, 20, 21].

(5) $$\frac{dP_{D}}{dt}=\rho_{P}\frac{dY}{dt}\;\Rightarrow \;P_{D}=\rho_{P}Y,$$

where *ρ*_*P*_ is the cell rate of expression of PD-1 on T cells. Again, following [18, 20, 21], the molar concentration of PD-L1 (*L*) within the tumor micro-environment is given by

(6) $$L=\rho_{L}(Y+\varepsilon T),$$

where *ρ*_*L*_ is the molar concentration of PD-1 per T cell and the parameter *ε* > 1 reflects the fact that the expression of PD-L1 is upregulated on tumor cells (and depends on the specific type of tumor). Finally, we choose the following functional form for T cell suppression via PD-1 signaling, *F*(*P*_*D*_, *L*), just as in [18, 20, 21] by

(7) $$F\left(P_{D},L\right)=\frac{1}{1+P_{D}L/K_{YQ}}.$$

The parameter values and their sources for the full pretreatment model are provided in Table 2.

### 2.2 | Model formulation with FGFR3, immune checkpoints, and combination therapy

**FIGURE 2:**
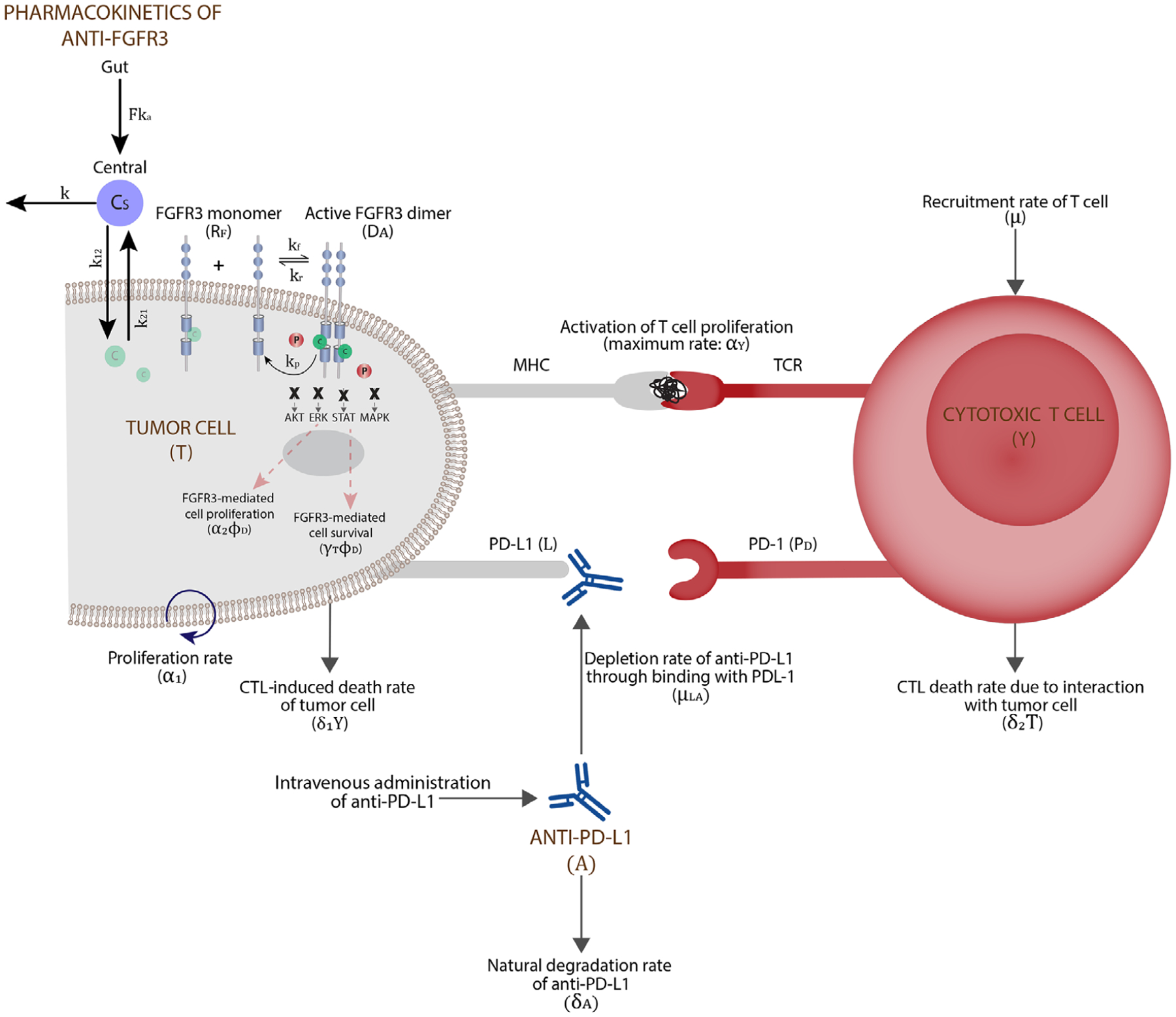
The microenvironment of a tumor cell with combination therapy consisting of a SMI of FGFR3 and an anti-PD-L1 antibody. We assume that the anti-FGFR3 drug binds with both FGFR3 monomers and dimers. The anti-PD-L1 antibody targets PD-L1, thus inhibiting its binding with PD-1 and enabling T cell activation and proliferation

In this section, we extend our pretreatment model equations to incorporate the therapeutic administration of an ICI in the form of a mAb against PD-L1 and a SMI targeting the FGFR3 pathway. We refer to the former as anti-PD-L1 therapy and the latter as anti-FGFR3 therapy, and our goal is to study the response of tumor cells to these therapies alone and in combination. (See Figure 2 for a schematic description of a tumor cell undergoing anti-FGFR3 and anti-PD-L1 combination therapy.)

An anti-PD-L1 antibody (*A*) binds to PD-L1 and inhibits the formation of the PD-1-PD-L1 complex. Following [18, 20, 21], the equation for the change in anti-PD-L1 antibody is given by:

(8) $$\frac{dA}{dt}=-\mu_{LA}LA-\delta_{A}A,$$

with an initial condition, *A*(0), that represents the amount of anti-PD-L1 antibody administered via intraperitoneal injection at different time points, *μ*_*LA*_ is the depletion rate of anti-PD-L1 antibody through binding with PDL-1 (*L*), and *δ*_*A*_ is the natural degradation rate of anti-PD-L1 antibody. Upon administration of an anti-PD-L1 antibody, the equation for the change in cytotoxic T cells (given in Equation 4) is modified and given by

(9) $$\frac{dY}{dt}=\left(\mu +\alpha_{Y}\frac{T}{\kappa +T}Y\right)F\left(P_{D},L,A\right)-\delta_{2}TY-\delta_{Y}Y.$$

The functional form *F*(*P*_*D*_, *L*, *A*) given by

(10) $$F\left(P_{D},L,A\right)=\frac{1}{1+\frac{P_{D}L}{K_{YQ}}\left(1-\frac{A}{A+K_{D}}\right)},$$

where *K*_*D*_ is the dissociation constant of the PD-L1/anti-PD-L1 complex. The factor *F*(*P*_*D*_, *L*, *A*) represents the impact of an anti-PD-L1 by reducing the number of PD-1/PD-L1 complexes within the tumor region. In the absence of an anti-PD-L1 antibody (i.e., *A* = 0), the factor *F*(*P*_*D*_, *L*, *A*) becomes *F*(*P*_*D*_, *L*) given by Equation (7). (See Appendix A for the full derivation of *F*(*P*_*D*_, *L*, *A*).)

By binding to the kinase activity region of the receptors, an anti-FGFR3 drug (rogaratinib) inhibits the phosphorylation of the FGFR3 kinase domain and the downstream signaling of protein kinase B (AKT), mitogen-activated protein kinase (MAPK), extracellular-signal-regulated kinase (ERK), and signal transducer and activator of transcription (STAT) [25–27]. To incorporate the therapeutic administration of rogaratinib, we designed a pharmacokinetic model with oral administration of rogaratinib. We assume that the tumor resides in a pharmacokinetic compartment of its own, and rogaratinib is transferred into the qaqatumor from the systemic circulation at the same rate as the peripheral tissue. The pharmacokinetics of rogaratinib and the system of equations (and all the underlying assumptions) governing the dynamics of FGFR3 in the tumor cell in the presence of rogaratinib are given in Appendices B and C, respectively.

Overall, the temporal dynamics of the tumor cells in the presence of combination therapy of anti-FGFR3 and anti-PD-L1 is given by

(11) $$\frac{dT}{dt}=\left(\alpha_{1}+\alpha_{2}\phi_{D}^{C}\right)T-\frac{\delta_{1}Y}{1+\gamma_{T}\phi_{D}^{C}}T\equiv P\left(T,\phi_{D}^{C}\right)-D\left(T,Y,\phi_{D}^{C}\right),$$

where $\phi_{D}^{C}$ is the fractional occupancy of active FGFR3 dimer per cell in the presence of anti-FGFR3 drug (described in Appendix A) and the temporal dynamics of cytotoxic T cells (*Y*) are given by Equation (9).

### 2.3 | Experimental studies and data

For mouse experiments, 6–8 week old female C57BL/6 mice were obtained from Jackson laboratory. Mice were housed in a specific pathogen-free animal facility at the University of Chicago. The MB49 cell line is a carcinogen-induced urothelial carcinoma cell line derived from a male C57BL/6 mouse, which was generously provided by Timothy L. Ratliff, Purdue University. The MB49-FGFR3G370C cell line was generated by retroviral transduction using the mammalian expression plasmid for internal ribosome entry site green fluorescent protein (pMXs-IRES-GFP) vector and sorted four times for GFP expression. For tumor growth experiments, mice were injected subcutaneously with 1 × 10^6^ MB49-FGFR3G370C tumor cells or GFP vector control MB49 tumor cells. Tumor volume was measured two times per week until the endpoint. Anti-PD-L1 antibody therapy was initiated when the tumor was first palpable. Mice were randomly assigned to intraperitoneal injection of either phosphate-buffered saline (PBS control) or anti-PD-L1 therapy (clone 10F.9G2; BioXcell). All experimental animal procedures were approved by the University of Chicago Animal Care and Use Committee (IACUC).

**FIGURE 3:**
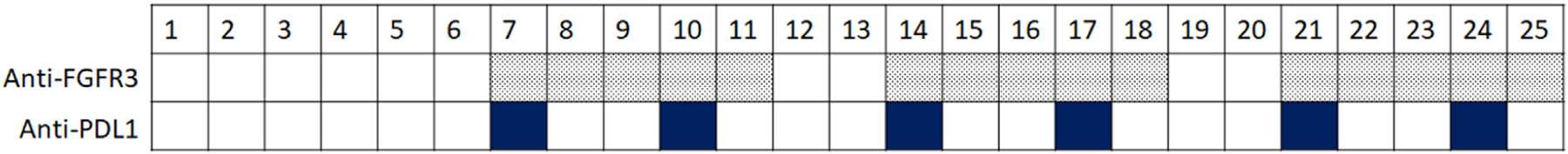
Dosing schedule of anti-FGFR3 and anti-PD-L1 monotherapies. We refer to this dosing schedule as the baseline schedule or Schedule 1

**FIGURE 4:**
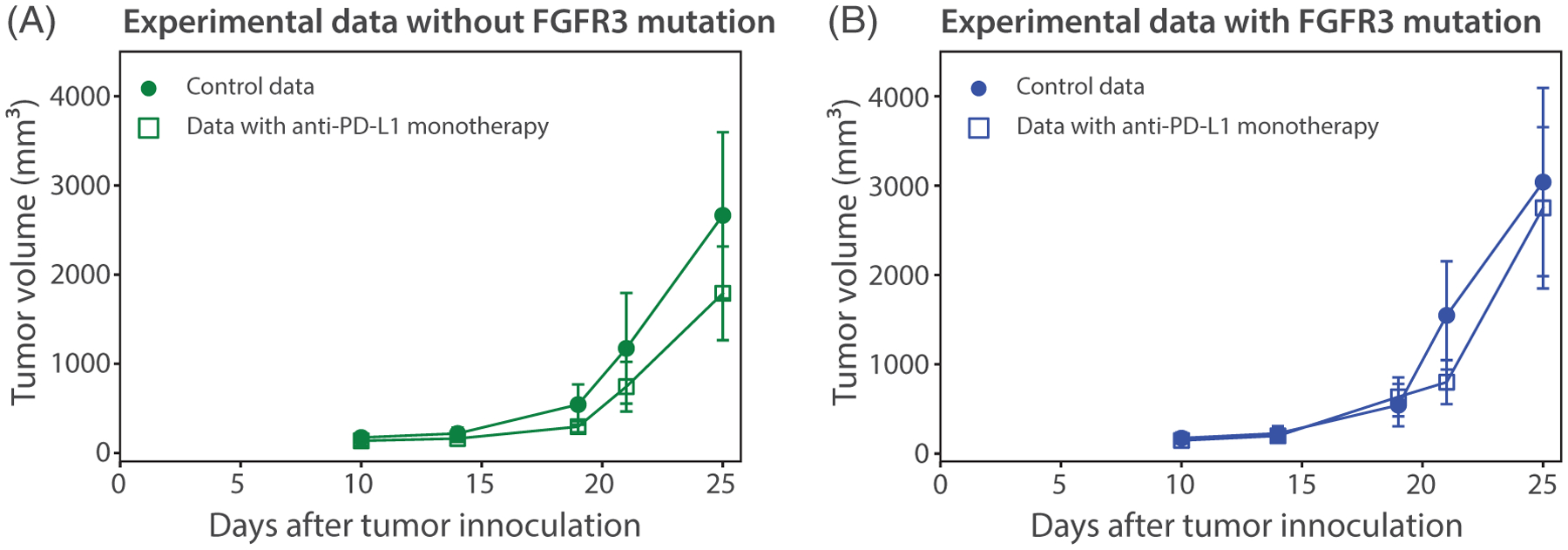
Plots showing (A) experimental data of tumor volume without FGFR3 mutation (circle: without treatment, square: with anti-PD-L1 monotherapy) and (B) experimental data of tumor volume with FGFR3 mutation (circle: without treatment, square: with anti-PD-L1 monotherapy). These experimental data show that mice without FGFR3 mutation receive more benefit from anti-PD-L1 therapy compared to mice with FGFR3 mutation

The dosing schedule for the therapies are presented in Figure 3: 75 mg/kg of the anti-FGFR3 drug is administered every day starting from day 7 through day 25 except on days 12, 13, 19, and 20 (these days are regarded as off-days) and 100 μg of anti-PD-L1 antibody is administered every three days starting on day 7 (except on the off-days). The anti-PD-L1 dosing scheme selected is based on preclinical dosing of antibodies targeting the PD-L1 immune checkpoint where patients are treated with anti-PD-L1 antibodies in an intermittent schedule given the prolonged half-life of immune checkpoint antibodies [28, 29]. For anti-FGFR3 therapy, there is data supporting intermittent dosing as well. Clinically, studies have given drug daily for 3 weeks, then taking 1 week off [30]. This strategy would be relatively in proportion to our intermittent dosing strategy in our mouse experiments. The experimental data of anti-PD-L1 monotherapy in tumors with and without FGFR3 mutation for time points 10, 14, 19, 21, and 25 are presented in Figure 4.

## 3 | PRETREATMENT RESULTS

### 3.1 | Parameter sensitivity

**FIGURE 5:**
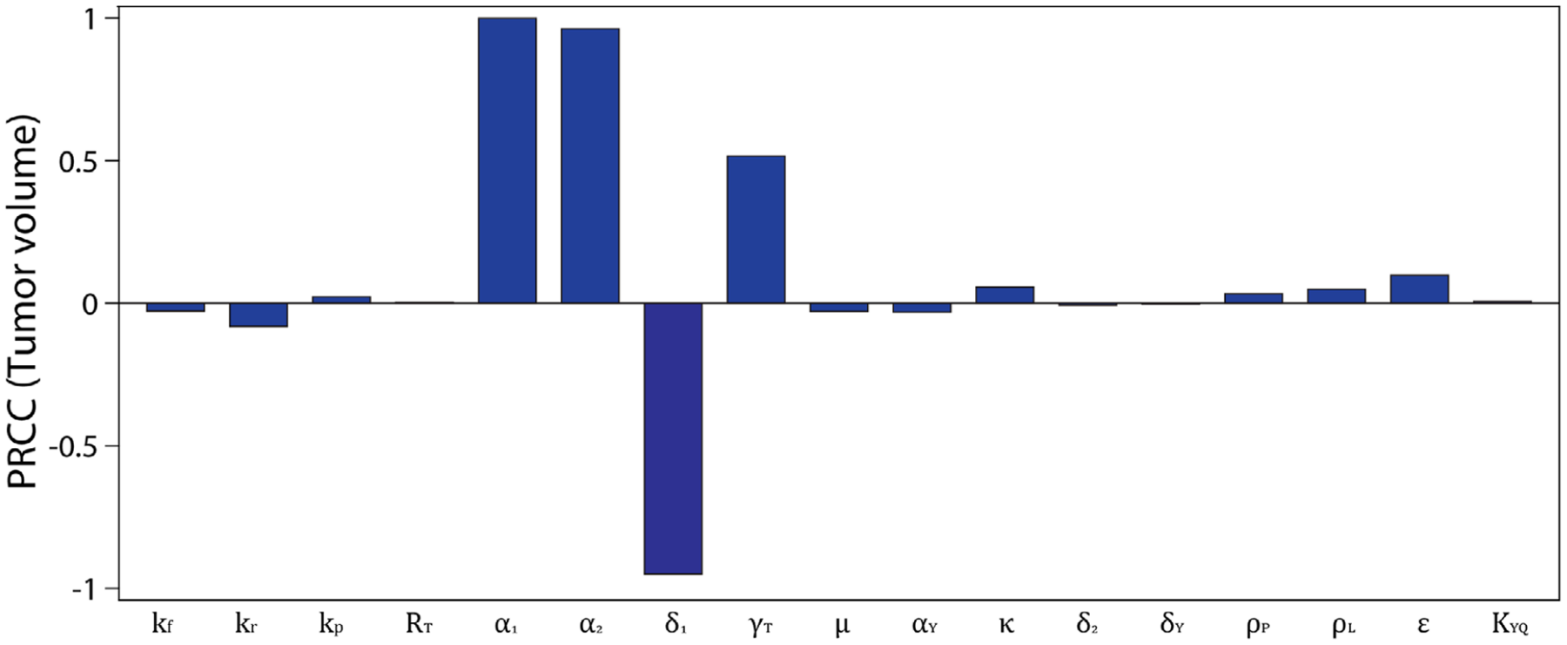
Sensitivity analysis for models (1), (3), and (4) showing PRCC values for the model parameters using the tumor volume as a response function. The baseline and range of parameter values used are given in Table 2

We use uncertainty and sensitivity analysis to determine the parameters that have the greatest effect on tumor growth in the FGFR3 mutation model without treatments (Equations 1, 3, and 4). Global sensitivity analysis quantifies the impact of the variations or sensitivity of each parameter of the model on the model outcomes [31–33]. In particular, following [32, 33], Latin hypercube sampling, and the partial rank correlation coefficient (PRCC) will be used for this analysis. The sensitivity analysis of the model is carried out using the tumor volume (in mm^3^) at the final time point, which is defined as $\frac{T\left(t_{f}\right)}{10^{6}}$, where *t*_*f*_ = 25 d. The range and baseline values of the parameters, tabulated in Table 2, will be used. The result depicted in Figure 5 shows that the parameters that significantly affect the tumor growth dynamics are the natural growth rate of tumor cells (*α*_1_), the CTL-mediated death rate of tumor cells (*δ*_1_), and FGFR3-mediated tumor proliferation (*α*_2_), and the sensitivity of tumor survival to FGFR3 (*γ*_*T*_).

### 3.2 | Pretreatment identifiability

To determine which model parameters, if any, can be uniquely estimated from a given dataset (and to what degree of certainty), we employ identifiability analysis [34]. This toolkit allows us to determine the subset(s) of identifiable parameters and explore their interplay without even using experimental data for parameter estimation and model calibration [35]. We examine both structural and practical identifiability of the model parameters.

#### 3.2.1 | Structural identifiability

First, we perform a structural identifiability analysis to determine whether or not it is possible to obtain a unique solution for the parameters while assuming perfect data (noise-free and continuous in time and space) [36–40]. Obtaining the parameter identifiability for nonlinear tumor-immune models is typically challenging [36]. In this section, we follow the approach in [36] and consider only the subset of the sensitive parameters identified in Section 3.1. We determine if these sensitive parameters can be uniquely estimated from measurements of values of all the model variables (active dimer complexes on tumor cells, tumor volume, and the number of cytotoxic T cells). The structural identifiability of the model is analyzed using the MATLAB package GenSSI (see [36, 39, 40] for complete details).

**FIGURE 6:**
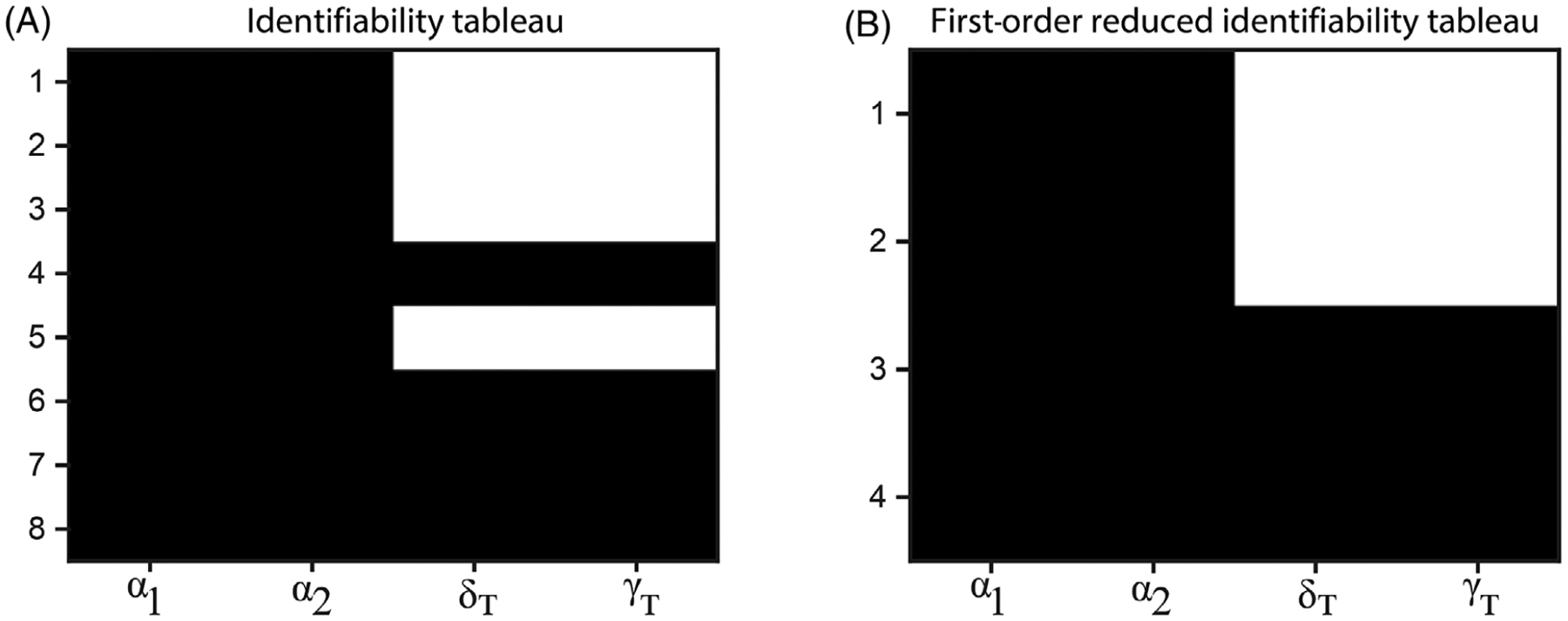
Identifiability results for the subset of the four most sensitive parameters of the model. (A) Identifiability tableau. (B) Results and reduced tableau. All four parameters shown are globally identifiable. Each row corresponds to an algebraic equation involving the parameters for which the cell in that row is black

We obtained an identifiability tableau in Figure 6A that shows eight nonzero rows—indicated by black regions—and corresponding to nonzero generating series coefficients—that depend on the sensitive parameters. If any parameters from the identifiability tableau can be computed as functions of the power series coefficients and eliminated, then a reduced tableau is obtained [36], as shown in Figure 6B. Using the GenSSI algorithm, we obtained unique solutions for all the sensitive parameters (*α*_1_, *δ*_1_, *α*_2_, *γ*_*T*_), that is, they are globally identifiable. Thus, the model is globally structurally identifiable, which indicates that error-free time series data of all the model variables would be sufficient to identify a unique subset of the four parameters.

#### 3.2.2 | Practical identifiability

In practice, complete time series and noiseless experimental data for structural identifiability are not available. Therefore, in this section, we carry out a practical identifiability analysis to determine whether the most sensitive parameters are identifiable from noisy experimental data of tumor volume. To do this, we seek to determine whether a distribution with a clear mode can be determined for each of the sensitive parameters given such data. We used the Markov chain Monte Carlo (MCMC) method with Metropolis–Hastings sampling [36]. Given simulated data for the system output, prior distributions of the parameter values, and a likelihood function, the MCMC samples the posterior distributions of the parameter, and the Metropolis–Hastings updating scheme accepts the new sample with probability given by the ratio of the new likelihood to the old likelihood [36].

Specifically, we use uniform distributions as prior distributions on the parameters within the ranges given in Table 2. To create the likelihood functions, we use the mean and standard deviation of the experimental data for tumor volume without FGFR3 mutation—to determine the practical identifiability of *α*_1_ and *δ*_1_—and the mean and standard deviation of the experimental data for tumor volume with FGFR3 mutation—to determine the practical identifiability of *α*_2_ and *γ*_*T*_. The tumor volume for each day is assumed to be log-normally distributed about the mean tumor volume at each time point and truncated to be within one standard deviation of this mean. The joint probability distribution of these is then used to create the likelihood functions for the two applications of the MCMC method. We first used MCMC to estimate the posterior distributions for *α*_1_ and *δ*_1_ and then separately used it for *α*_2_ and *γ*_*T*_. In both cases, we used a chain length of 10,000 to sample from the posterior distributions.

**FIGURE 7:**
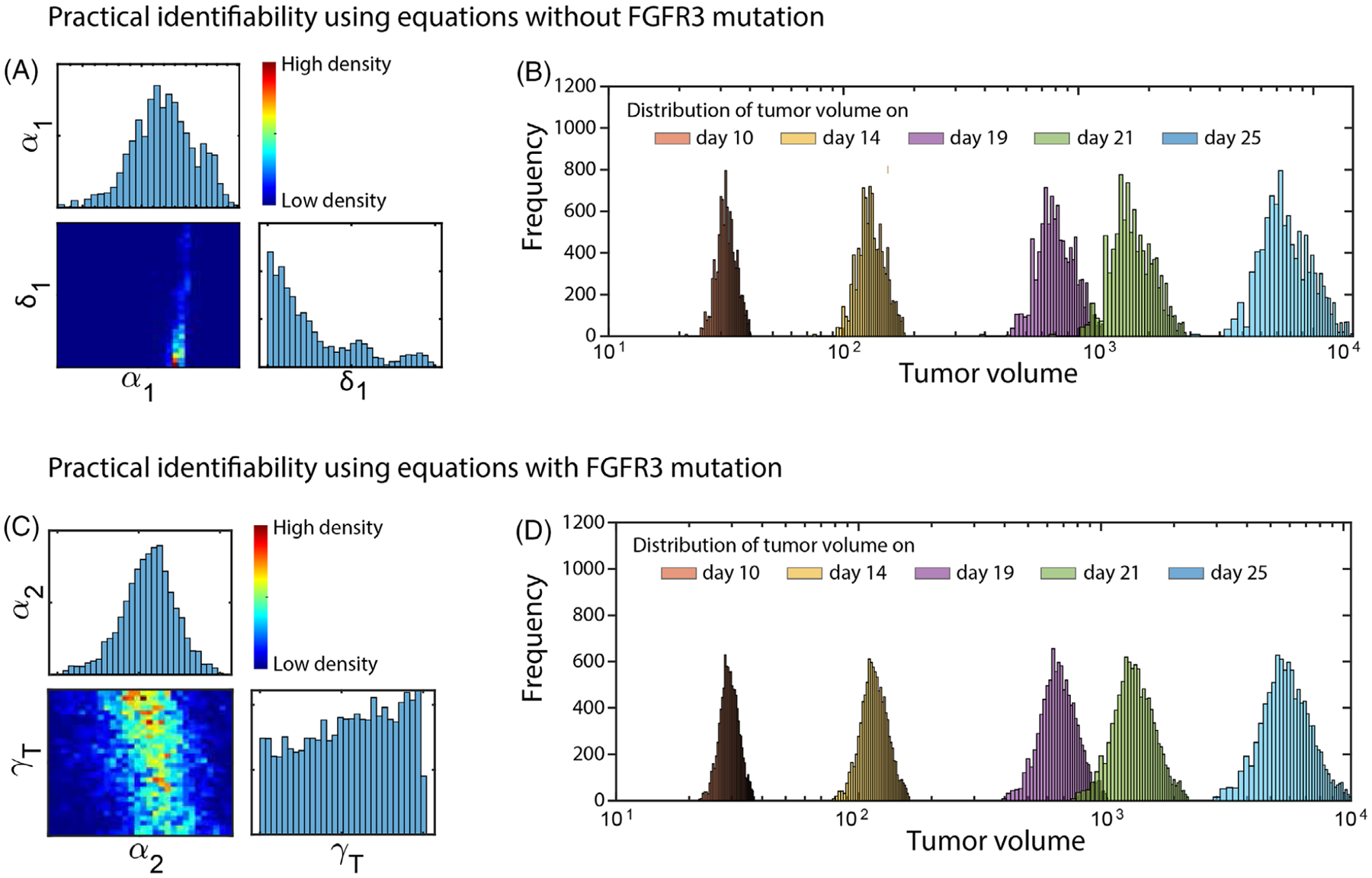
(A) Matrix of two-dimensional heat maps with one-dimensional histograms on the diagonal showing the parameter distributions of *α*_1_ and *δ*_1_ using experimental data of tumor volume without FGFR3 mutation. (B) The distributions of tumor volume for the MCMC chain, for five time points using experimental data of tumor volume without FGFR3 mutation. (C) Matrix of two-dimensional heat maps with one-dimensional histograms on the diagonal showing the parameter distributions of *α*_2_ and *γ*_*T*_ using experimental data of tumor volume with FGFR3 mutation. (D) The distributions of tumor volume for the MCMC chain, for five time points using experimental data of tumor volume with FGFR3 mutation

The result depicted in Figure 7A in the form of one-dimensional histograms and two-dimensional heat maps shows that *α*_1_ has a normal distribution and the likelihood-based confidence region of *δ*_1_ is infinitely extended in decreasing direction in its range in Table 2, thus indicating that *α*_1_ is practically identifiable and *δ*_1_ is not practically identifiable (although the likelihood has a unique minimum for *δ*_1_) [39]. Then, by sampling from this posterior distribution and forward simulating, we generate model predictions of tumor volume distributions without FGFR3 mutation at the sample time points that are tightly controlled and follow the mean ± SD of the corresponding data on days 14, 19, 21, and 25 (Figure 7B). This result suggests that data on days 14, 19, 21, and 25 may be ideal for estimating the identifiable parameter *α*_1_. (See Figure D-1 in Appendix D for comparisons between the predicted distribution and the experimental data.) Using experimental data for mean tumor volume with FGFR3 mutation, our simulation showed that *α*_2_ and *γ*_*T*_ have a normal and a broad distribution, respectively (Figure 7C), within its range in Table 2. Hence, *α*_2_ is practically identifiable and *γ*_*T*_ is not practically identifiable given the available experimental data [36]. We again sample from the posterior distribution and forward simulate to generate tumor volume distributions with FGFR3 mutation, and again we see that these distributions are tightly controlled and follow the mean ± SD of the corresponding data on days 14, 19, 21, and 25 (Figure 7D) indicating that the data on time points 14, 19, 21, and 25 may be ideal for estimating the identifiable parameter *α*_2_. (See Figure D-1 in Appendix D for comparisons between the predicted distribution and the experimental data.) It is important to note that practical nonidentifiability is generally a result of the insufficient amount or quality of the available experimental data [36, 39, 40]; thus, it is possible to resolve the practical nonidentifiability of *δ*_1_ and *γ*_*T*_ with additional tumor volume experimental data.

### 3.3 | Pretreatment parameter estimation

**FIGURE 8:**
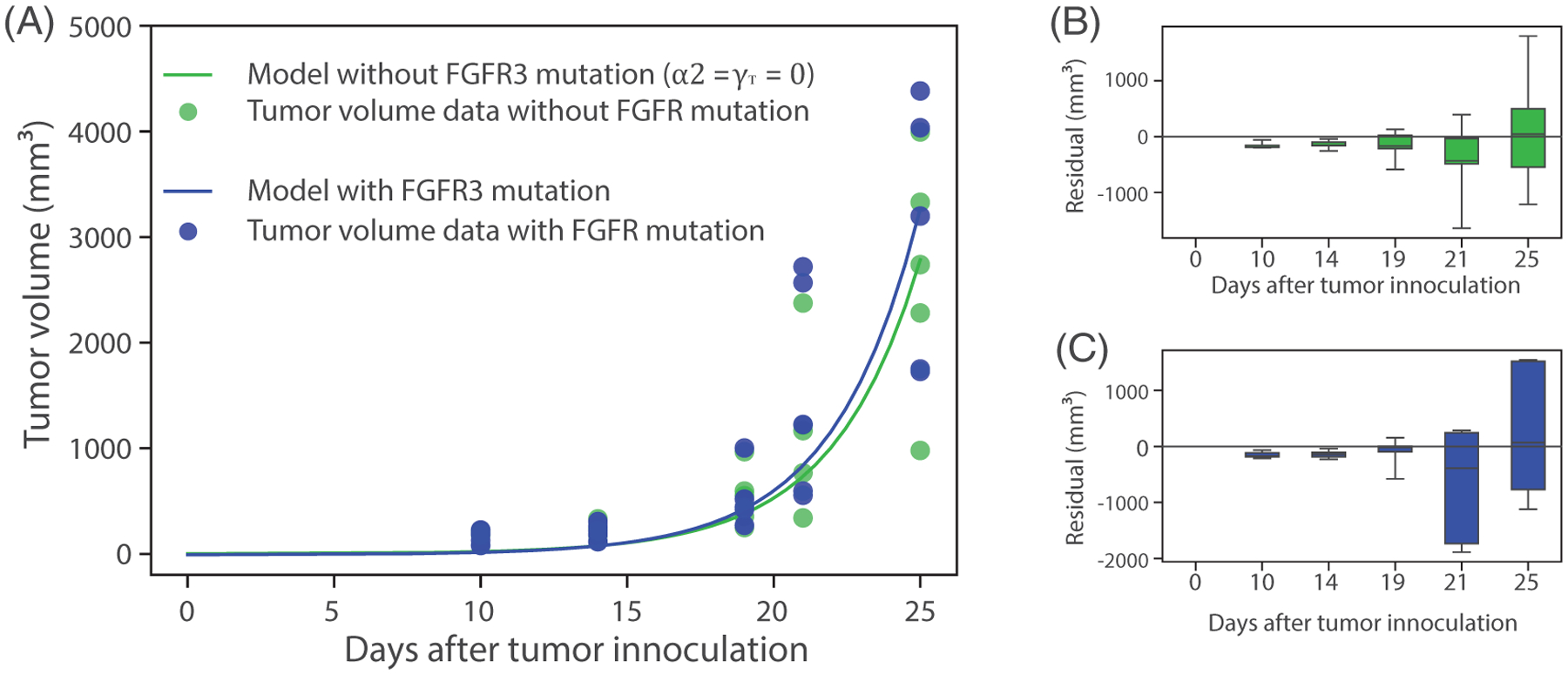
(A) Green curve: Calibration of models (3) and (4) with *α*_2_ = *γ*_*T*_ = 0 to the experimental data of tumor without FGFR3 mutation in mice (*n* = 5) to estimate the FGFR3-independent growth rate (*α*_1_ = 0.337 d^−1^ (95% CI: 0.33–0.3439)). Blue curve: Calibration of models (1), (3), and (4) to the growth curve of tumor with FGFR3 mutation in mice (*n* = 5) to estimate FGFR3-dependent parameters (*α*_2_ = 0.00774 d^−1^ (CI: 0.0072–0.01) and *γ*_*T*_ = 0.3018 (CI: 0.3–0.304)). (B, C) Residual plots showing that the models predict tumor volume without FGFR3 mutation and with FGFR3 mutation, respectively

Having determined the identifiability properties of the most significant parameters both structurally and practically, we turn to estimating these parameters from experimental data. Specifically, we fit the mathematical model to two growth curves of MB49 bladder cancer cell lines, with and without mutant FGFR3 as described above. We use experimental data of tumor volume versus time (five time points) for five mice without mutant FGFR3 to estimate the FGFR3-independent tumor growth rate (*α*_1_). We use the MATLAB **lsqcurvef it** function with ode15s solver (with relative and absolute tolerances of **tol** = 10^−10^), and an initial condition, given by *T*(0) = 10^6^ cells and *Y*(0) = 3.2 × 10^5^ cells [19], to carry out the data-fitting process. By calibrating the only unknown parameter (*α*_1_) in our model without the FGFR3 mutation (*α*_2_ = *γ*_*T*_ = 0) in Equation (3) to the experimental data (Figure 8A. green curve), we obtained the best fit value for *α*_1_ = 0.337 d^−1^ (95% CI: 0.33 − 0.3439), which corresponds to a bladder tumor doubling time of 2.1 days in mice. This is in line with previously reported tumor growth rates [41–43]. The box plot of the residual vector shown in Figure 8B indicates that the model can accurately predict temporal changes tumor volume in mice without the FGFR3 mutation. We note that we get the same best fit value for *α*_1_ = 0.337 d^−1^ when the model without FGFR3 mutation is calibrated with the last four time points of experimental data from which we saw better fits from our identifiability analysis (Section 3.2.2, Figure D-1).

With FGFR3-independent parameters estimated, we next calibrate the model with FGFR3 mutation (Equations 1, 3, and 4). Specifically, we use experimental data of tumor volume versus time when the FGFR3 mutation is present in mice (Figure 8A, red curve) to estimate two parameters associated with ligand-independent activation of FGFR3 (i.e., the FGFR3-mediated tumor proliferation rate (*α*_2_ = 0.00774 d^−1^, 95% CI: 0.0072–0.01) and the FGFR3-mediated survival sensitivity parameter (*γ*_*T*_ = 0.3018, 95% CI: 0.3–0.304). As before, we generated box plots of residuals (Figure 8C), indicating that the model can accurately predict tumor volume when the FGFR3 mutation is active. It is important to note that growth of the experimental tumor cell line is not dependent on the FGFR3 activating mutation, which was exogenously introduced. Thus, we do not expect the FGFR3 activating mutation to have a significant impact on tumor growth as observed in both the data and model simulation.

### 3.4 | Relative impact of FGFR3-dependent pathways on tumor growth

**FIGURE 9:**
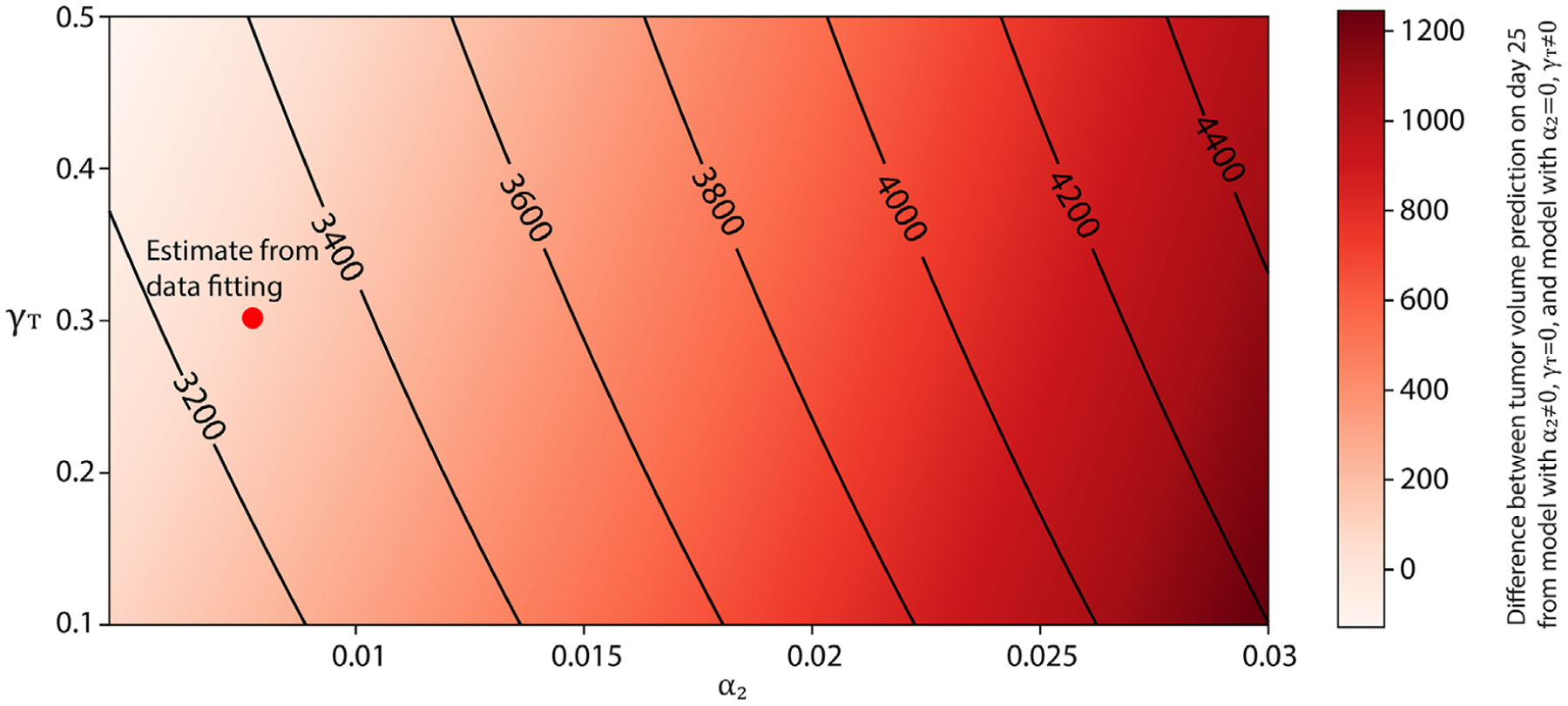
Heatmap showing the difference between tumor volume in mice on day 25 predicted from model with *α*_2_ ∈ [0.001, 0.03] and *γ*_*T*_ = 0 and volume in mice on day 25 predicted from model with *α*_2_ = 0 and *γ*_*T*_ ∈ [0.1, 0.5]. The red dot represents the difference between the model with *α*_2_ = 0.00774 d^−1^, *γ*_*T*_ = 0 and the model with *α*_2_ = 0 d^−1^, *γ*_*T*_ = 0.3018, indicating that FGFR3 mutation has an almost equal effect on both tumor proliferation and survival in the experimental design. The contour plot shows the prediction of tumor volume on day 25 at the different pairs of *α*_2_ and *γ*_*T*_ in the ranges [0.001,0.03] and [0.1,0.5], respectively

With all parameters associated with tumor growth now estimated, an important question arises about which FGFR3-mediated effect, increased proliferation or increased survival, independently results in a greater measurable increase in tumor volume. There are potential clinical applications for determining the contribution of proliferation and apoptosis independently on tumor growth. For instance, there are inhibitors of apoptosis such as BCL-2-targeted drugs, which could be investigated in a context where apoptosis is more aberrantly regulated. We investigated this by estimating, from simulations, the difference between the tumor volume on day 25 when the FGFR3 survival benefit is switched off (i.e., *α*_2_ ∈ [0.001, 0.03] and *γ*_*T*_ = 0) and when the FGFR3 proliferative benefit is turned off (i.e., *α*_2_ = 0 and *γ*_*T*_ ∈ [0.1, 0.5]). In this way, we compare their relative contributions to tumor growth, and in Figure 9 we see the parameter space by which the FGFR3-dependent pathways lead to more tumor growth. It is interesting to note that the region in parameter space that corresponds to the proliferation effect resulting in larger tumors is much more expansive. This region also contains the point corresponding to our estimated parameters as shown by the red dot in Figure 9, though the difference in the effect on tumor volume there is slight.

The results in this section are complementary to those presented in the section on parameter sensitivity, as that analysis does not tease out the impact on tumor growth when the FGFR3 survival/proliferation benefit is completely nonexistent. By investigating the knockdown of the proliferative and survival benefit independently, we are able to better understand how a completely effective therapy that specifically targets apoptosis versus proliferation would impact tumor reduction. Additionally, these findings may have implications for fine tuning the antitumor effect in a way that mitigates detrimental effects on T cells or other immune cells. If the relative components of apoptosis to proliferation differ between immune cells and tumor cells, then this difference could be exploited to improve the therapeutic index of cancer treatments.

## 4 | TREATMENT RESULTS

We next turn to the question of understanding the effects of therapy on the tumor reduction. Specifically, in this section, we simulate the model with immune checkpoint and FGFR3-targeted therapy alone and in combination.

### 4.1 | Treatment with anti-PD-L1 antibody alone

**FIGURE 10:**
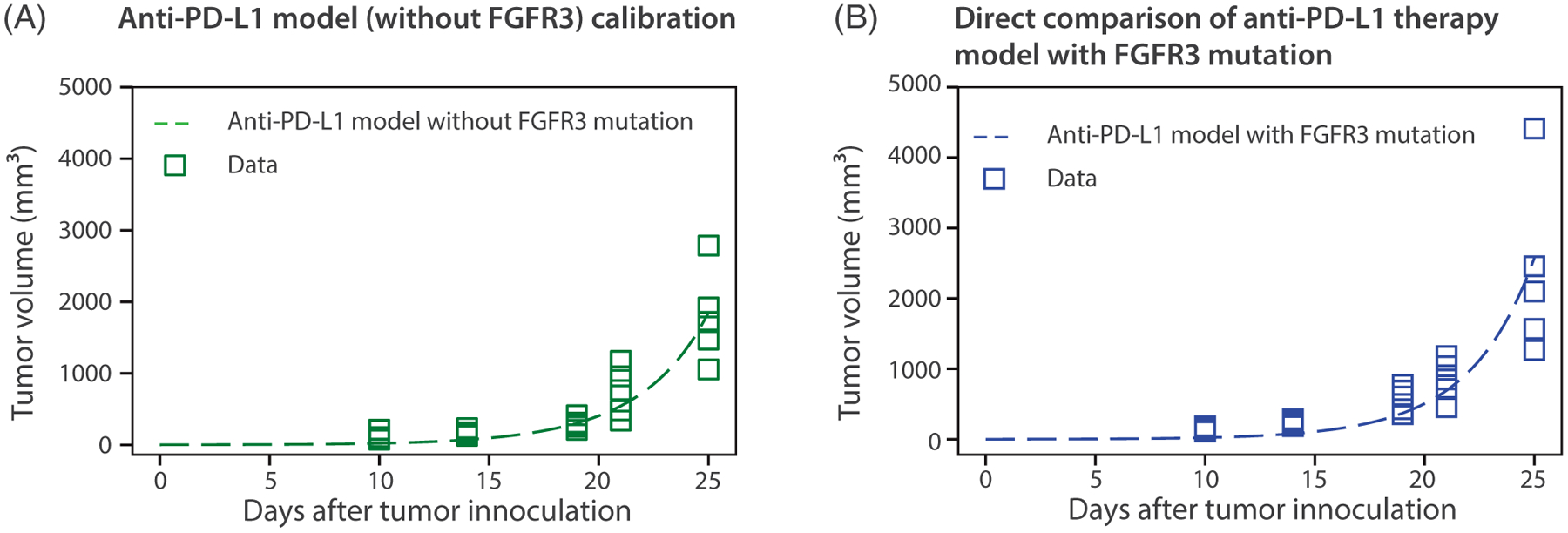
(A) Anti-PD-L1 therapy model calibration to experimental data of anti-PD-L1 therapy on tumor cells without FGFR3 mutation to estimate *K*_*D*_ = 0.1005 nM, *μ*_*LA*_ = 2.6611 × 10^−5^ d^−1^. (B) The estimated parameters are used to simulate the anti-PD-L1 therapy model with FGFR3 mutation and directly compared with the corresponding data in mice. Parameter values used are given in Tables 2 and 3

In order to study the effect of monotherapy with an anti-PD-L1 immunotherapeutic agent, we calibrated the anti-PD-L1 therapy model without FGFR3 mutation (i.e., Equations 3, 8, and 9 with *α*_2_ = *γ*_*T*_ = 0) with the experimental data for anti-PD-L1 therapy on tumor cells without the FGFR3 mutation in mice, to estimate the antibody dissociation constant (*K*_*D*_) and the depletion rate of anti-PD-L1 antibodies through binding to PD-L1 (*μ*_*LA*_). The anti-PD-L1 antibody has a half-life of 2 days, thus, the natural degradation rate of the anti-PD-L1 is *δ*_*A*_ = ln(2)∕2 ≈ 0.3466 d^−1^. We used the MATLAB **lsqcurvef it** function with ode15s solver for calibration. These results are shown in Figure 10A and Table 3. With the model now calibrated to data where the FGFR3 mutation is absent, we turn to validating the model with data where the FGFR3 mutation is active. Specifically, to accomplish this validation step, we directly compared (i.e., no additional parameter fitting) the simulation of the anti-PD-L1 therapy model with FGFR3 mutation (i.e., Equations 1, 3, 8, and 9) with the corresponding experimental data. The result in Figure 10B shows an excellent correlation between the model and the data without the need for parameter tuning.

**FIGURE 11:**
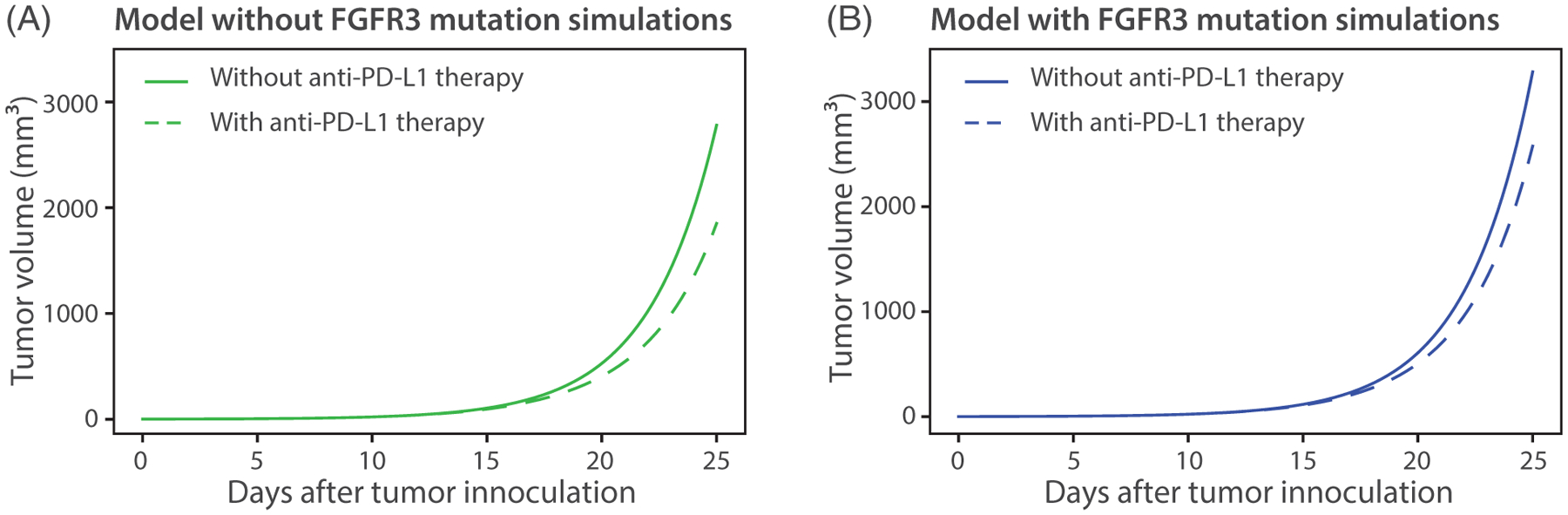
Comparison of models with anti-PD-L1 therapy. Results in (A) and (B) show that mice without FGFR3 mutation receive more benefit from anti-PD-L1 therapy compared to mice with FGFR3 mutation. Parameter values used are given in Tables 2 and 3

In Figure 11, the model output from Figures 10A and B is compared to the corresponding models without anti-PD-L1 therapy. The larger gap in Figure 11A compared to Figure 11B shows that mice without FGFR3 mutation receive more benefit from anti-PD-L1 therapy compared to mice with FGFR3 mutation. The data generated specifically for this study and reported in Figure 4 supports this finding.

### 4.2 | Treatment with anti-FGFR3 inhibitor alone

**FIGURE 12:**
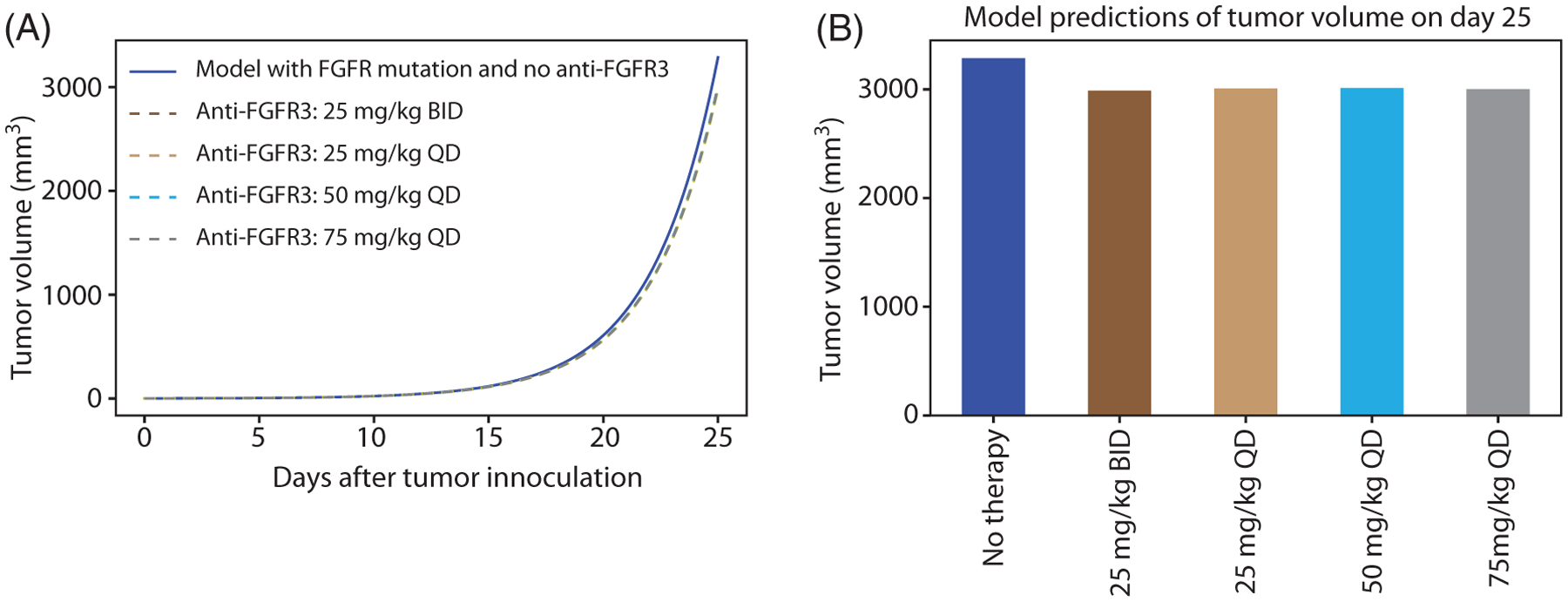
Simulation of anti-FGFR3 therapy model with FGFR3 mutation with no treatment, 25 mg/kg QD, 25 mg/kg BID, 50 mg/kg QD, and 75 mg/kg QD of rogaratinib using the dosing schedule in Figure 3. (B) Model prediction of tumor volume on day 25 in mice. Parameter values used are given in Tables 2, B-1, and C-1

Next, we investigate targeted therapy against the FGFR3 receptor using the dosing schedule in Figure 3. To estimate rogaratinib pharmacokinetic parameters, we fit a three-compartment model for rogaratinib biodistribution (described in Appendix B) to experimental data of rogaratinib plasma concentration in mice [25]. Using these parameter values, we simulated (see Figure 12) FGFR3 mutant tumor response to the following doses of rogaratinib : 25 mg/kg QD (once a day), 25 mg/kg BID (twice a day), 50 mg/kg QD, and 75 mg/kg QD using the dosing schedule in Figure 3. It is clear from Figure 12 that the various doses of anti-FGFR3 drugs do not have substantial impacts on the tumor volume. Also, the effect sizes of the doses are approximately equal. These results are not surprising since this tumor cell line is not dependent on the FGFR3 activating mutation—which was exogenously introduced to study its impact on anti-PD-L1 therapy as shown in Figure 11—for enhanced tumor growth.

### 4.3 | Treatment with combination therapy

Treatment with anti-PD-L1 and anti-FGFR therapy each have proven efficacy in bladder cancer independently and are currently used [4, 8]. This section considers combination anti-FGFR therapy and anti-PD-L1 therapy, which is an active area of investigation and has shown promise in early reports [45]. Below, we use the mathematical model to make testable therapeutic predictions at a time when there have been no published experimental studies with combination FGFR and immune checkpoint targeted therapy.

**FIGURE 13:**
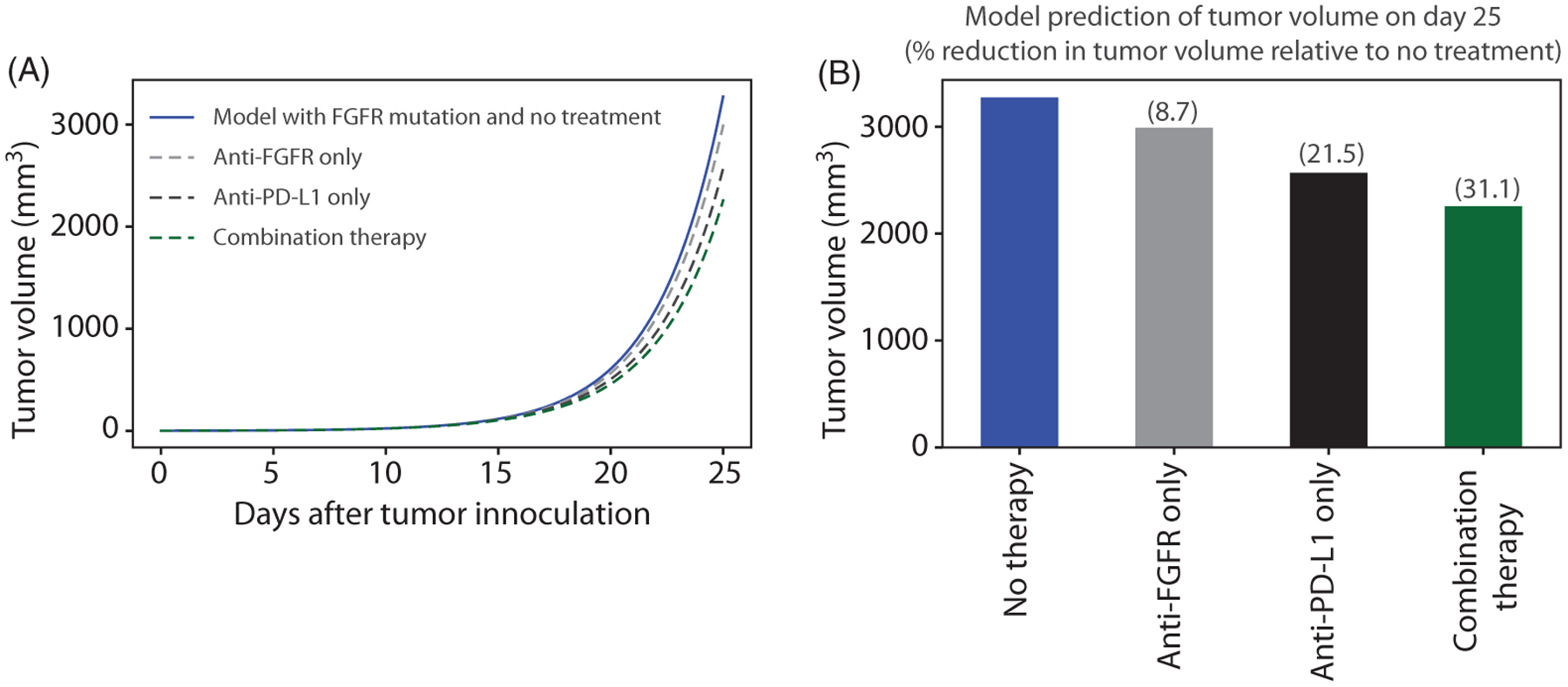
(A) Simulation of the model with FGFR3 mutation with no therapy (blue), anti-FGFR3 therapy only (gray), anti-PD-L1 therapy only (black), and combination therapy (green). (B) Model prediction of tumor volume on day 25 in mice. Parameter values used are given in Tables 2–C-1

We simulated the model to predict the effect of combining anti-FGFR3 and anti-PD-L1 therapies on tumor cells with FGFR3 mutation in mice (using the dosing schedule in Figure 3 with cotreatment on days 7, 10, 14, 17, 21, and 24). The result is shown in Figure 13, along with the impact of anti-FGFR3 therapy only and anti-PD-L1 therapy only. Our model predictions show that the effect of each therapy is approximately additive when combined, and combination therapy reduces the tumor volume on day 25 by 33.3% compared to 21.9% in the case of anti-PD-L1 therapy only. Similar results were obtained when the anti-PD-L1 therapy is combined with either 25 mg/kg QD, 25 mg/kg BID, or 50 mg/kg QD dose of rogaratinib (results not shown).

**FIGURE 14:**
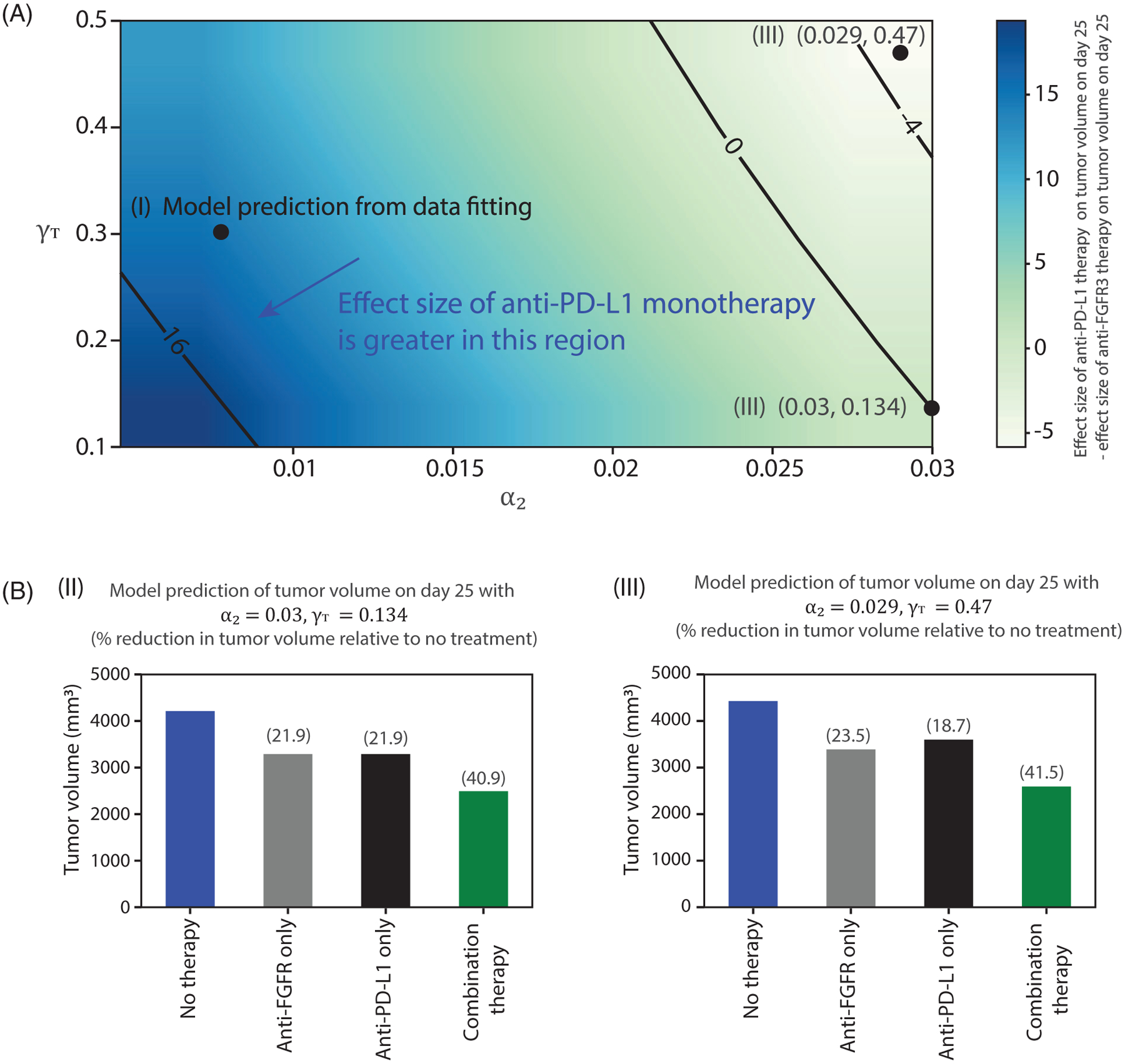
(A) A heatmap showing the difference in effect size of anti-PD-L1 and anti-FGFR3 therapies on day 25 on tumors with FGFR3 mutation. The black dot (i) indicates an example where anti-PD-L1 therapy has a larger effect size than anti-FGFR3 therapy (the values for the effect sizes are shown in Figure 13B). (B) Prediction of tumor volume and effect size of anti-PD-L1 monotherapy, anti-FGFR3 monotherapy, and combination therapy using (ii) *α*_2_ = 0.03 d^−1^ and *γ*_*T*_ = 0.134 where the effect size of anti-PD-L1 and anti-FGFR3 are approximately equal; and (iii) *α*_2_ = 0.029 d^−1^ and *γ*_*T*_ = 0.47 where anti-PD-L1 therapy has lesser effect size than anti-FGFR3 therapy. The arrow indicates the direction of increasing relative strength of anti-PD-L1 monotherapy

We further simulated the model with a wider range of the parameters that govern FGFR3 impact on proliferation (*α*_2_ ∈ [0.001, 0.03]) and survival (*δ*_1_ ∈ [0.1, 0.5]) to compare the effectiveness of anti-PD-L1 and anti-FGFR3 monotherapies when the influence of the FGFR3 mutation on tumor growth varies. The results depicted in Figure 14 show that for some combinations of *α*_2_ and *γ*_*T*_, especially in the region where the FGFR3 pathway has a significant impact on tumor growth, the targeted therapy outperforms the immune checkpoint monotherapy (this result also shows the possible significant impact of anti-FGFR3 monotherapy on FGFR3 overexpressing cancers). It is also important to note, by comparing Figure 13B to Figure 14 BII, that the efficacy of combination therapy can be significantly increased in parameter ranges where there is a substantial increase in the effectiveness of rogaratinib while anti-PD-L1 therapy retains its efficacy.

### 4.4 | Kaplan–Meier survival analysis

Kaplan–Meier survival analysis is used to measure the fraction of subjects living for a certain amount of time after treatment in an experiment or clinical trial [46]. To further estimate the effects of anti-FGFR3 monotherapy, anti-PD-L1 monotherapy, and combination therapies on the tumor with FGFR3 mutation using the baseline dosing schedule in Figure 3, we carried out a Kaplan–Meier analysis by measuring the fraction of mice, *S*_*t*_, surviving at time *t* using the formula given below:

(12) $$S_{t}=\frac{N-N_{TV\geq 2000\;{\text{mm}}^{3},t}}{N},$$

where *N* is the total number of mice and $N_{TV\geq 2000\;{\text{mm}}^{3},t}$ is the number of mice that did not survive (i.e., mice with tumor volume (*TV*) above or equal to the survival threshold (2000 mm^3^)) at time *t*. We simulated 50 mice by first choosing their sensitive parameters (*α*_1_, *δ*_*T*_, *α*_2_, and *γ*_*T*_) randomly within their ranges of values given in Table 2. In particular, we used normal sampling distributions for practically identifiable parameters (*α*_1_ and *α*_2_) using their respective mean and standard deviation and uniform sampling distributions for practically nonidentifiable parameters (*δ*_1_ and *γ*_*T*_). We then repeated the batch of 50 simulations four more times for a total of five n silico experiments with each using the same method for randomly choosing the four sensitive parameters.

**FIGURE 15:**
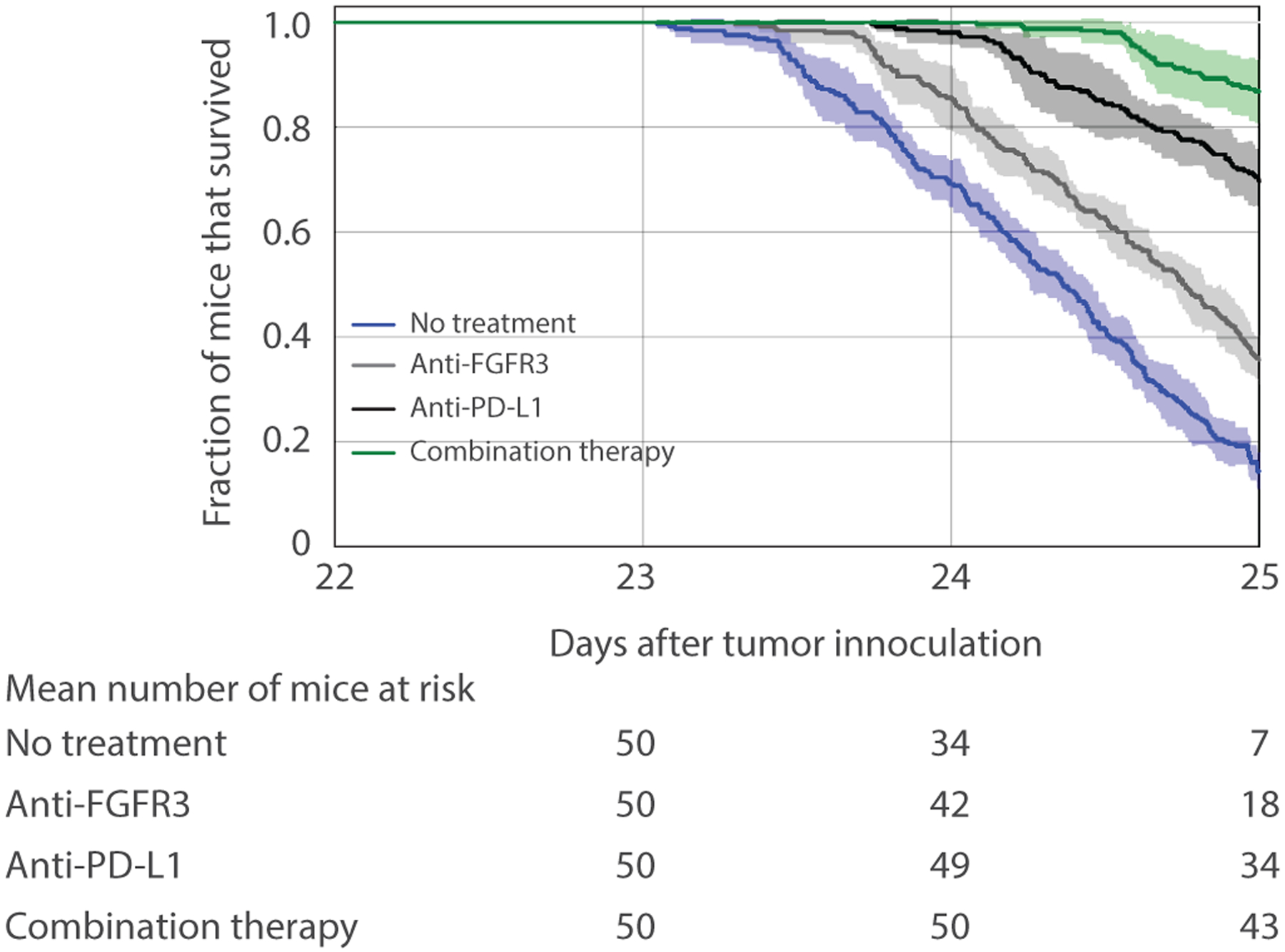
Kaplan–Meier survival analysis showing the fraction (mean ± SD) of mice that survived with no treatment (blue), anti-FGFR3 monotherapy (gray), antiPD-L1 monotherapy (black), and combination therapy (green). Parameter values used are in Tables 2–C-1 with normal distribution of *α*_1_ (with mean = 0.337 d^−1^ and standard deviation = 0.0034 d^−1^), normal distribution of *α*_2_ (with mean = 0.00774 d^−1^ and standard deviation = 0.0016 d^−1^), uniform distributions of *δ*_1_ and *γ*_*T*_ within their range of values in Table 2

The result depicted in Figure 15 shows that 78–96% of the mice treated with combination therapy survived on day 25 compared to mice treated with anti-PD-L1 monotherapy (62–76%), anti-FGFR3 monotherapy (28–42%), or untreated mice (10–20%). Since the values for the FGFR3-dependent parameters are within the region where anti-PD-L1 monotherapy has more effect size than anti-FGFR3 monotherapy (Figure 14A), we expect that more mice would survive when treated with anti-PD-L1 therapy.

**FIGURE 16:**
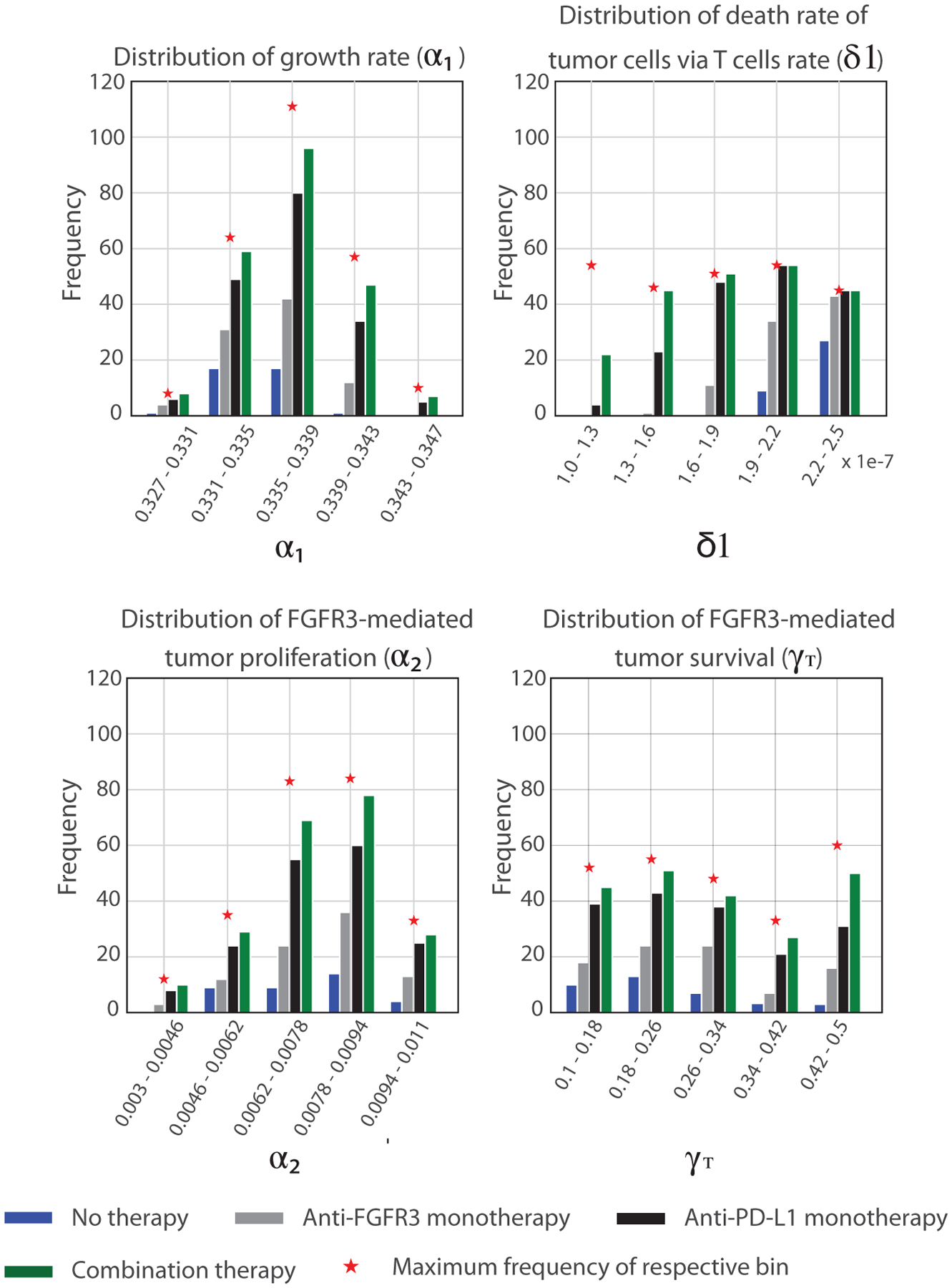
Distribution of sensitive parameters related to mice that survived on day 25 of survival analysis with different therapeutic conditions. The distances between each bar and the maximum frequency represent the distribution of mice that did not survive in the Kaplan–Meier analysis

Figure 16 shows the distribution of parameters associated with the mice that survived until day 25 in the Kaplan–Meier survival analysis. These results show that the surviving mice are characterized by ahigh CTL-induced death rate (*δ*_1_) (i.e., slow-growing tumor cells). In particular, the only untreated mice that survived until day 25 had a CTL-induced death rate above 1.9 × 10^−7^ cell^−1^ d^−1^; those treated with anti-FGFR3 monotherapy needed at least a value of 1.6 × 10^−7^ cell^−1^ d^−1^ (with one exception). Even anti-PD-L1 monotherapy and combination therapy needed *δ*_1_ to exceed 1.3 × 10^−7^ cell^−1^ d^−1^ to give at least even odds for the mice to survive until day 25.

The Kaplan–Meier survival analysis presented above consists of exploratory numerical simulations conducted with the goal of making biologically testable predictions at a time when there have been no published experimental studies with combination anti-FGFR therapy and anti-PD-L1 therapy, though preclinical investigations are ongoing. It is encouraging that these results are concordant with experimental data and emerging clinical data [45].

### 4.5 | Dosing schedules

**FIGURE 17:**
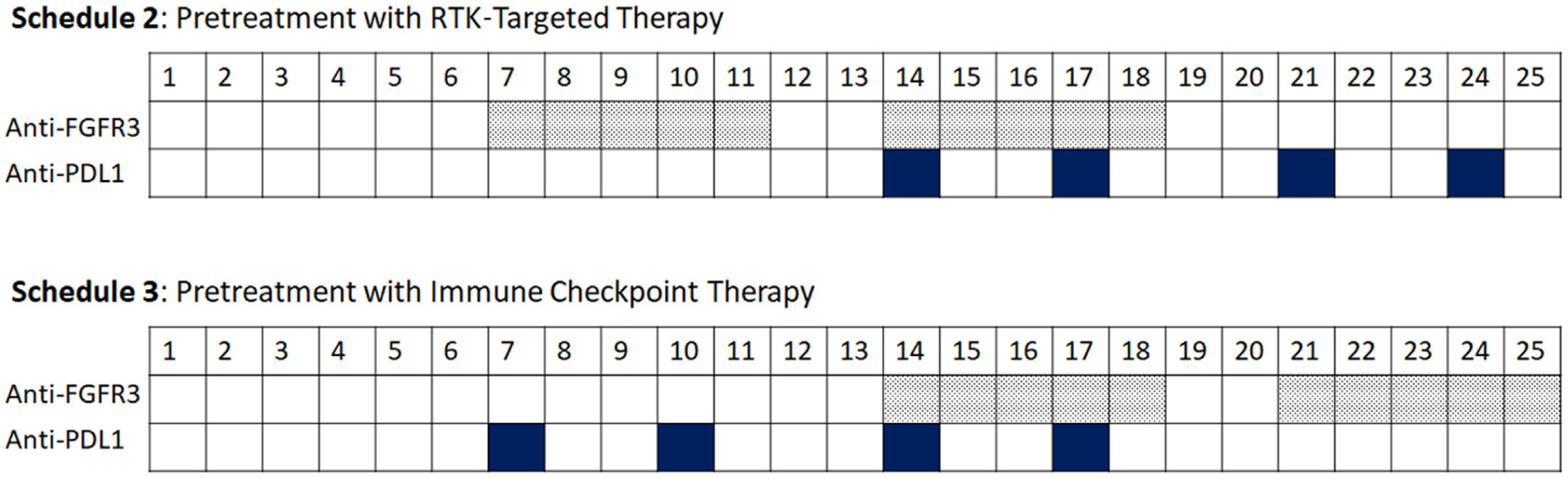
Dosing schedule of pretreatment with anti-FGFR3 therapy and pretreatment with anti-PD-L1 therapy

Next, we use the model to determine how best to administer anti-PD-L1 and anti-FGFR3 targeted therapies. To determine the most favorable combinations and to investigate the potential synergy between anti-PD-L1 and anti-FGFR3 therapies in mice with FGFR3 mutation, we simulate different dose scheduling for anti-FGFR3 and anti-PD-L1 therapies (Figure 17). There have been no published studies with combination anti-FGFR therapy and anti-PD-L1 therapy, though studies are ongoing. A major goal of these simulations is to determine the strategy for combining these two therapies that best optimize efficacy. Furthermore, anti-FGFR therapy (erdafitinib) is FDA approved for use after chemotherapy, but it remains unknown whether it should be given before or after anti-PD-L1 immune checkpoint therapy. Thus, determining an optimal sequence remains an important clinical question.

In these simulations, we considered treatments of tumor cells with FGFR3 mutation with a total of 10 doses of 75 mg/kg QD of rogaratinib (based on prior work characterizing this drug [25]) and four doses of 100 μg of anti-PD-L1 antibody using the dosing schedules 2 and 3 shown in Figure 17 (compared to 15 doses of anti-FGFR3 and six doses of anti-PD-L1 in the dosing schedule (baseline schedule) in Figure 3).

**FIGURE 18:**
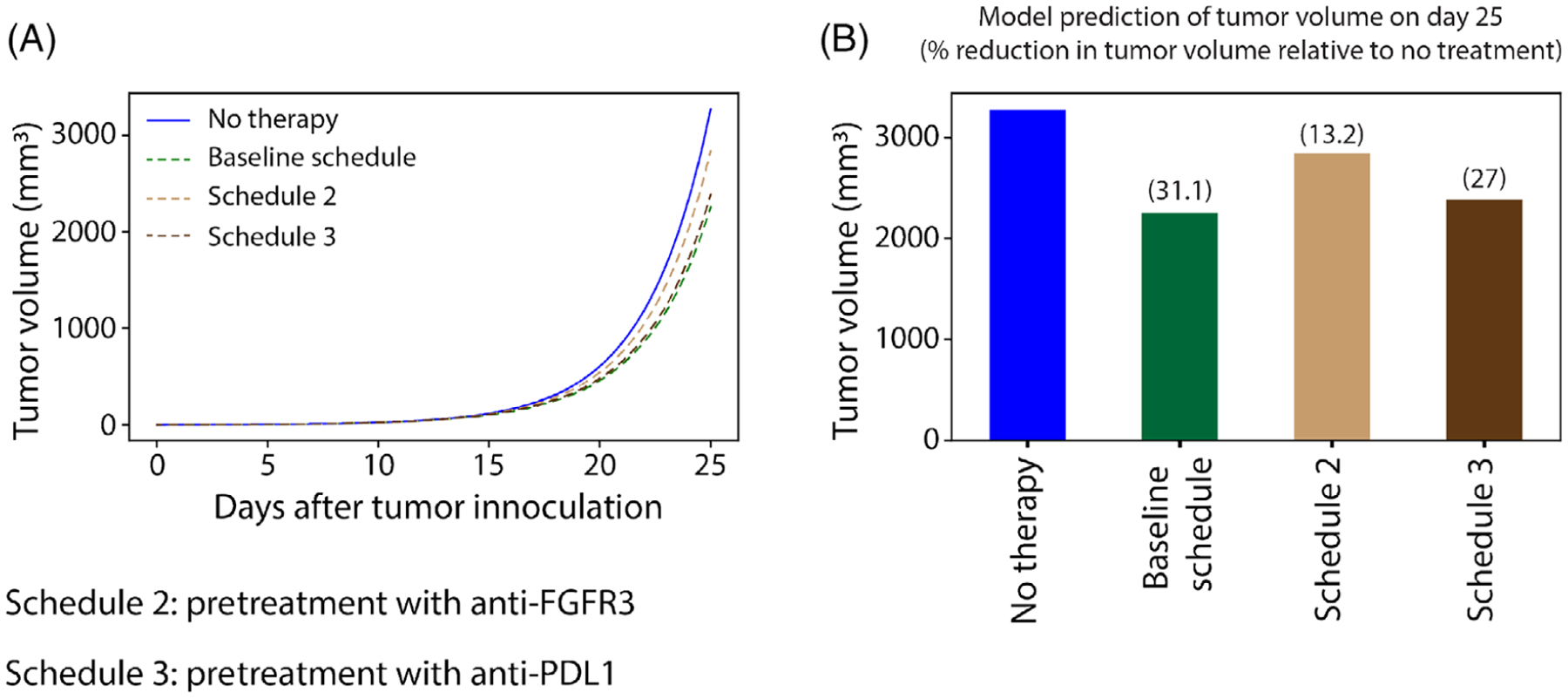
Simulation showing effect size of the different dosing schedules. (A) Simulation of the model with FGFR3 mutation with no therapy (blue), treatment with baseline combination therapy (green), pretreatment with anti-FGFR3 therapy, and pretreatment with anti-PD-L1 therapy. (B) Model prediction of tumor volume on day 25 in mice. Parameter values used are given in Tables 2–C-1

**FIGURE 19:**
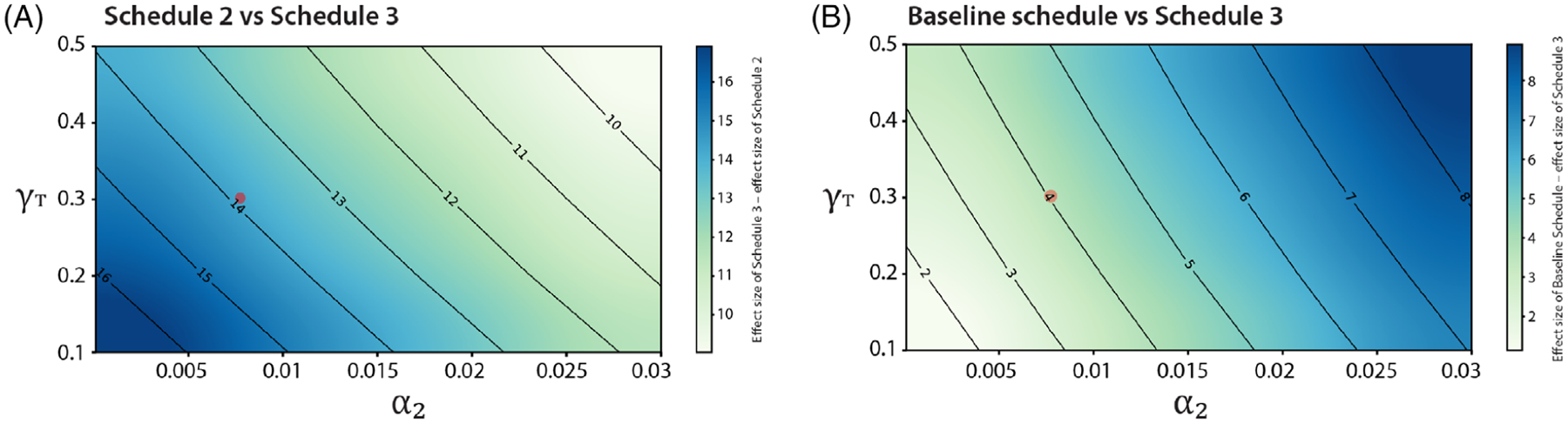
Simulation showing comparison between the effect size of (A) Schedule 2 versus Schedule 3 (B) Baseline schedule versus Schedule 3. Parameter values used are given in Tables 2–C-1 with *α*_2_ ∈ [0.001, 0.03] and *γ*_*T*_ ∈ [0.1, 0.5]

The ultimate goal is to determine the optimal dosing strategy that minimizes tumor growth while also minimizing the amount of drug administered. The tumor is either pretreated with anti-FGFR3 therapy or pretreated with anti-PD-L1 therapy as shown in Figure 18. The results depicted in Figures 18 show that the pretreatment of tumors with anti-PD-L1 therapy (Schedule 3) is more effective than the pretreatment of tumors with anti-FGFR3 therapy (Schedule 2). This result persists throughout the *α*_2_ − *γ*_*T*_ parameter space, even in regions where anti-FGFR3 monotherapy greatly outperforms immune checkpoint monotherapy (Figure 19A). It is also important to note that the outcomes for Schedule 3 are compared to those from the baseline schedule of cotreatment (as shown in Figure 19B), which administers five additional doses of anti-FGFR3 therapy and two additional doses of the anti-PD-L1 antibody.

## 5 | DISCUSSION

Cisplatin-based chemotherapy is the standard of care, and until recently, nearly the only recourse for people suffering from advanced bladder cancer (Stages II–IV) [47, 48]. Outcomes remained discouraging as many patients either fail to respond to treatment or suffer recurrent disease within 5 years [49, 50]. After nearly four decades of little progress, immunotherapy with checkpoint inhibitors (PD-L1 and PD-1) has fundamentally shifted the treatment paradigm of bladder cancer [50]. At the same time, advances in the understanding of the molecular biology of bladder cancer have led to the identification of molecular pathways, such as FGFR3 signaling, upon which new therapeutic approaches can be targeted [51]. In this paper, we developed an experimentally validated mathematical model for the dynamics of advanced bladder cancer growth and response to receptor tyrosine kinase (RTK) targeted therapy alone and in combination with an ICI. This model is the first of its kind in that it incorporates the molecular details of an FGFR3 mutation that initiates signaling via ligand-independent dimerization to enhance tumor cell proliferation and survival. Our model formulation allows us to track the fraction of active FGFR3 dimers and to use this quantity to augment the rates of tumor cell division and tumor cell death, which is mediated by cytotoxic T cells. A second important feature of our model is that it explicitly accounts for the formation of PD-1/PD-L1 complexes that inhibit T cell proliferation and activation.

The model is carefully calibrated and validated with experimental measures of tumor volume with and without the FGFR3 mutation. Global sensitivity analysis revealed four parameters that have the greatest influence on tumor volume. The parameter sensitivity results indicate that therapies (monotherapies or combination therapies) that reduce the natural growth rate of tumor cells, increase the death rate of tumor cells by cytotoxic T-cells (e.g., the use of antibodies to target the immune checkpoint PD-1/PD-L1 pathway to active cytotoxic T-cells), and/or decreasing fractional occupancy of FGFR3 dimer complexes on tumor cells (e.g., the use of anti-FGFR3 drugs to target the FGFR3 pathway) will be effective in controlling and treating bladder cancer with FGFR3 mutation. In an attempt to identify which FGFR3-mediated effect has more impact on tumor growth, we computed the difference between the tumor volume when FGFR3 only impacts the tumor cell proliferation rate and the tumor volume when FGFR3 only impacts tumor cell survival. The results suggest that FGFR3 mutation can lead to increased tumor volume primarily due to either proliferation or survival effects—depending on the relative strengths of these signaling pathways, that is, the parameters. However, the proliferation effect is more influential across a larger region of parameter space. Interestingly, for our estimated parameter values, the effects of FGFR3 on proliferation and survival are nearly equal.

Based on the mechanisms of action of an ICI targeting PD-L1 and a tyrosine kinase inhibitor targeting FGFR3 (rogaratinib), we extended our model to evaluate the impact of these therapies alone and in combination. Simulations of anti-PD-L1 therapy showed that tumors without the FGFR3 mutation are more susceptible to anti-PD-L1 therapy than tumors with the FGFR3 mutation. This effect is likely independent of FGFR3 effects on intrinsic tumor growth and survival, since both cell lines grow essentially at the same rate in the presence or absence of FGFR3 activating mutations. These results are in line with our reported experimental data in Figure 4 and suggest that the FGFR3 mutation can impact the effectiveness of anti-PD-L1 therapy. Furthermore, the experiments described here use a tumor cell line that is not dependent on the FGFR3 activating mutation, which was exogenously introduced. Thus, we did not expect the FGFR3 activating mutation to have a significant impact on tumor growth. Our anti-FGFR3 monotherapy model simulations clearly show that this is indeed the case for four different doses of rogaratinib. However, when we simulated a wider range of the parameters that govern FGFR3 impact on proliferation and survival, we saw that for realistic values of *α*_2_ and *γ*_*T*_, anti-FGFR3 therapy can not only have a substantial impact on tumor reductionbut targeted therapy can actually outperform anti-PD-L1 monotherapy.

Despite the slight impact of rogaratinib monotherapy on tumor cells with FGFR3 mutation when baseline parameters are used, our model simulations show that its combination with anti-PD-L1 therapy increases the effect size of the anti-PD-L1 therapy on tumor cells with the FGFR3 mutation. That is, while anti-PD-L1 antibody loses efficacy when the FGFR3 mutation is active, anti-PD-L1 antibody impact on tumor reduction is recovered when combined with a drug that targets FGFR3. In fact, Kaplan–Meier survival analysis showed that when mice with FGFR3 mutant bladder cancer are treated with combination therapy, they have a much higher probability of surviving to day 25 compared to mice treated with either monotherapy. We also found that there are parameter ranges for of *α*_2_ and *γ*_*T*_ where there is a significant increase in tumor reduction due to rogaratinib and only a small decrease in tumor reduction due to immune checkpoint therapy, and this leads to a substantial increase in the efficacy of combination therapy.

In an attempt to find the most effective way of delivering combinations of these two therapies, we simulated two different dose-scheduling regimens for rogaratinib and an ICI targeting PD-L1. We compared outcomes of these strategies to each other and to our baseline dose schedule of cotreatment, which administers five additional doses of rogaratinib. Our results show that pretreatment with anti-PD-L1 therapy leads to greater tumor reduction than pretreatment with anti-FGFR3 therapy. Interestingly, even in parameter regimes where anti-FGFR3 monotherapy greatly outperforms immune checkpoint monotherapy, the model predicts that it is still better to pretreat with the anti-PD-L1 drug. Furthermore, our baseline schedule of cotreatment performs only slightly better, with five additional doses of anti-PD-L1 therapy, than pretreatment with anti-PD-L1 therapy. This result suggests that some patients may benefit more from pretreatment with anti-PD-L1 because fewer drug doses can be used to achieve similar outcomes. These findings have direct clinical relevance given that anti-FGFR3 therapy is currently FDA approved, but it remains unknown whether it is best employed prior to or after anti-PD-L1 immunotherapy.

This modeling study not only quantifies the influence of the FGFR3 mutation on bladder cancer growth; it also predicts various outcomes for RTK and ICI mono- and combination therapy. In the current model formulation, we are considering the total amount of FGFR3 monomers in the system and allowing all monomers to interact with each other. The resulting dimerization of monomers allows us to quantify the temporal changes in fractional occupancy of active FGFR3 dimers in the system and their impact on tumor growth dynamics. In future iterations of the model, we could relax these assumptions and reformulate the model so that FGFR3 monomers only interact with other monomers on the same cell. We are currently modifying this model to describe different mechanisms of immune cell kill. We will also extend the model to include the impact of spatial dynamics by translating this system of ordinary differential into an agent-based modeling framework. Continued computational modeling of bladder cancer therapy can potentially lead to patient-specific optimization of a combination of anti-FGFR3 with anti-PD-L1 treatments.

## Figures and Tables

**TABLE 1:** Description of variables

Variable	Description	Units
*R*	Free FGFR3 monomer receptors	nmol
*D*	Active FGFR3 dimer complexes	nmol
*T*	Tumor cells	cells
*Y*	Cytotoxic T cells (CTLs)	cells
*P*_*D*_	PD-1	nM
*L*	PD-L1	nM

**TABLE 2:** Pretreatment parameter values

Variable	Description	Range of values (baseline)	Units	Source
FGFR3 related				
*k*_*f*_	FGFR3 association rate	(1–5) × 10^11^ (4.16 × 10^11^)	nmol^−1^ d^−1^	[22]
*k*_*r*_	FGFR3 dissociation rate	10–2000 (864)	d^−1^	[22]
*k*_*p*_	FGFR3 recycling rate	10–150 (112.32)	d^−1^	[22]
*R*_*T*_	Total FGFR3 receptors	(1.49–1.74) × 10^−11^ (1.66 × 10^−11^)	nmol cell^−1^	[23]
Tumor related				
*α*_1_	Intrinsic proliferation rate	0.12–0.51 (0.337)	d^−1^	Best fit
*α*_2_	FGFR3-mediated proliferation rate	0.001–0.1 (0.00774)	d^−1^	Best fit
*δ*_1_	CLT-mediated death rate	(1–2.5) × 10^−7^ (1.1 × 10^−7^)	cell^−1^ d^−1^	[19]
*γ*_*T*_	FGFR3-enhanced survival sensitivity	0.1–0.5 (0.3018)		Best fit
T cell related				
*μ*	Activation/recruitment rate	(1–2) × 10^4^ (1.3 × 10^4^)	cell^−1^ d^−1^	[19]
*α*_*Y*_	Maximum proliferation rate	0.1–0.5 (0.3044)	d^−1^	[19, 20, 24]
*κ*	Proliferation half-saturation constant	10^6^ to 3 × 10^7^(2.019 × 10^7^)	cell	[19]
*δ*_2_	Tumor-mediated death rate	(2–4) × 10^−10^ (3.422 × 10^−10^)	cell^−1^ d^−1^	[19]
*δ*_*Y*_	Natural death rate	0–0.05 (0.0412)	d^−1^	[18]
*ρ*_*P*_	PD-1 per cell	10^−9^–10^−7^ (1.258 × 10^−8^)	nM	[18]
*ρ*_*L*_	PD-L1 per cell	10^−9^ to 2 × 10^−7^ (2.51 × 10^−8^)	nM	[18]
*ε*	Tumor - immune PD-L1 ratio	1–100 (50)		[18, 20, 24]
*K*_*YQ*_	Immune checkpoint inhibition constant	10^−4^–10^−2^ (1.296 × 10^−3^)	nM^2^	[18]

**TABLE 3:** Parameter values for anti-PD-L1 antibody

Parameter	Description	Value	Units	Reference
*μ*_*LA*_	Anti-PD-L1 depletion via binding to PD-L1	2.66 × 10^−5^	nM^−1^ d^−1^	Best fit
*δ*_*A*_	Anti-PD-L1 natural degradation rate	0.3466	d^−1^	Computed from half-life
*K*_*D*_	PD-L1-anti-PD-L1 dissociation constant	0.1005	nM	Best fit*

* The dissociation constants of various anti-PD-L1 antibodies with PD-L1 can vary over several orders of magnitude with typical values ranging between 0.0467 and 1.75 nM [44]. Our value of 0.1005 nM is in line with these values.

**FIGURE B. 1:**
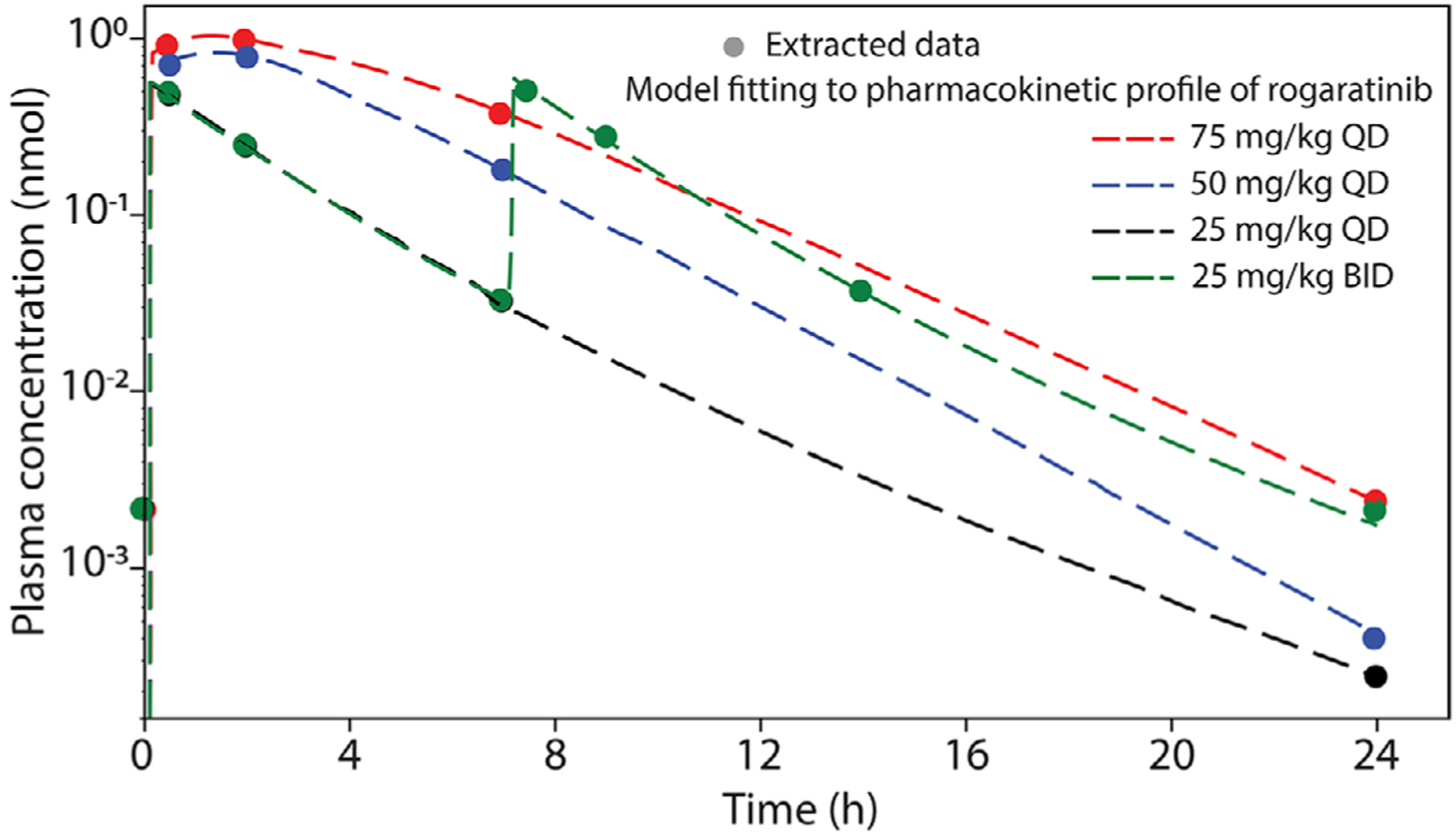
Time profiles of single-dose of 75 mg/kg QD (red dashed line), 50 mg/kg QD (blue dashed line), 25 mg/kg QD (black dashed line), and 25 mg/kg BID (green dashed line) of rogaratinib in plasma. The best fit of the model is plotted together with experimental data of rogaratinib in mice described in [25]. Parameter values used are given in Tables 2–C-1

**FIGURE C. 1:**
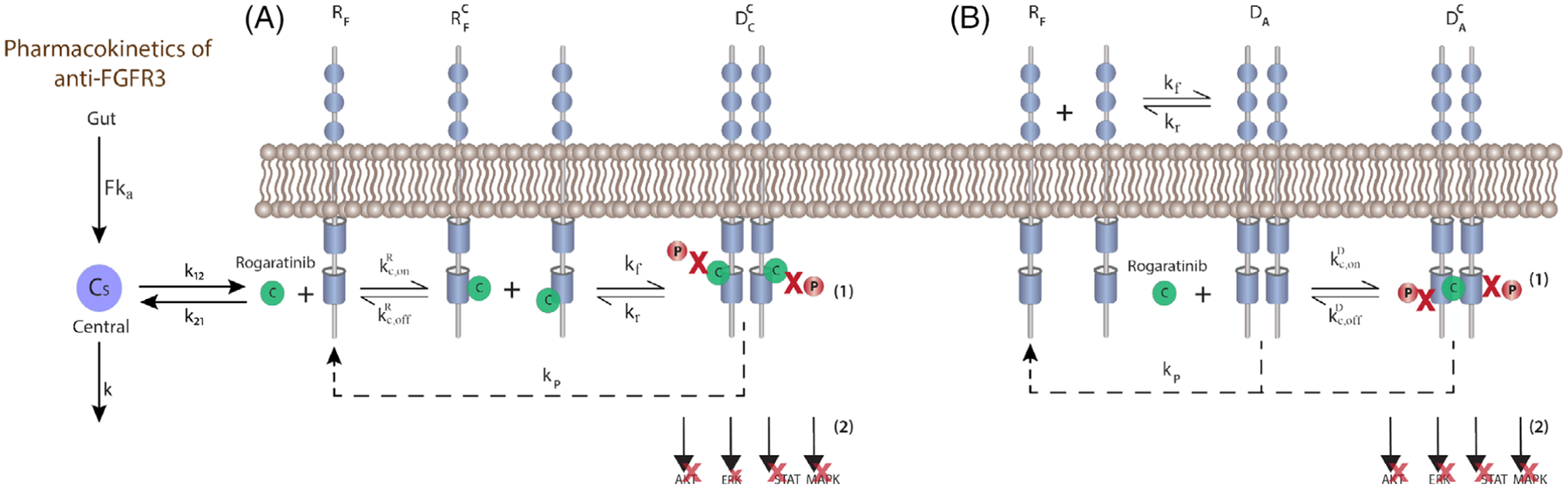
Flowchart of rogaratinib as anti-FGFR3 treatment. (A) Rogaratinib drug associated with FGFR3 monomer on tumor cells at a rate $k_{C,on}^{R}$ to form $R_{F}^{C}$ and dissociate at a rate $k_{C,off}^{R}$. $R_{F}^{C}$ dimerize at a rate $k_{f}^{C}$ to form $D_{C}^{C}$ which can either dissociate at rate *k*_*r*_, or internalize and recycled into FGFR3 monomer at a rate *k*_*p*_. (B) Rogaratinib binds with an active dimer *D*_*A*_ at a rate $k_{C,on}^{D}$ to form $D_{A}^{C}$ which dissociate at a rate $k_{C,off}^{D}$. These events lead to (1) inhibition of FGFR3 phosphorylation; and consequently, (2) inhibition of downstream signaling of AKT, MAPK, ERK, and STAT

**FIGURE D. 1:**
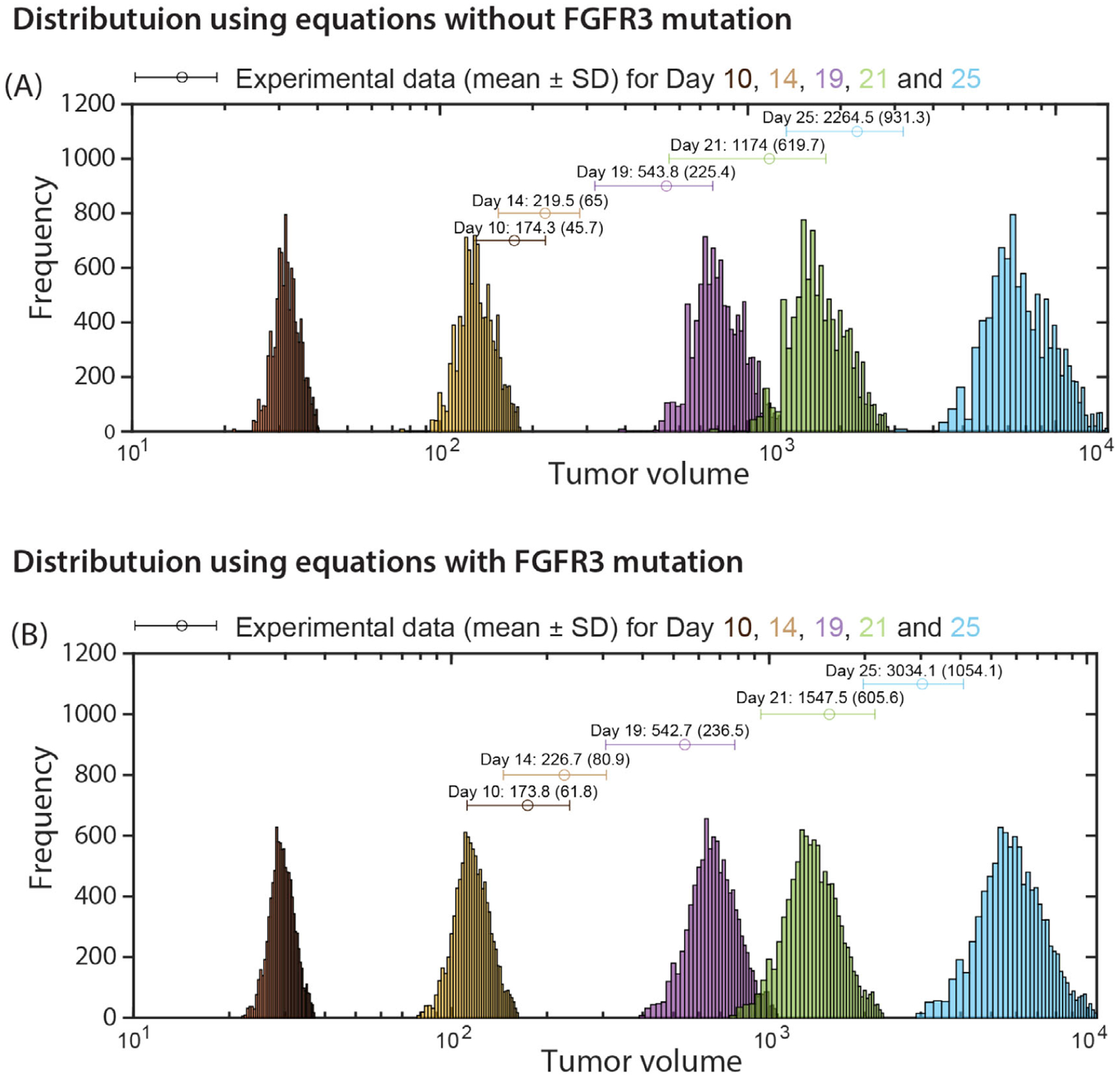
(A) Comparison of the predicted distributions of tumor volume without FGFR3 mutation for the MCMC chain with experimental data. (B) Comparison of the predicted distributions of tumor volume without FGFR3 mutation for the MCMC chain with experimental data. Both figures show that model distributions match the corresponding data distribution on days 14, 19, 21, and 25

## APPENDIX A

### A.1 | Formulation for PD-1/PD-L1 complexes,

*Q* Following [20], the molar concentration of PD-L1 (*L*) within the tumor microenvironment consists of the amount of free PD-L1 and the amount of PD-L1 bound to the drug,

$$L=L_{\text{free}}+L_{\text{bound}},$$

with *L* = *ρ*_*L*_(*Y* + *εT*). We consider the following reaction:

$$P_{D}+L_{\text{free}}\underset{\delta_{Q}}{\overset{\alpha_{PL}}{⇌}}Q,$$

Following [18, 20, 21, 24], we assume that the association and dissociation of *Q* are fast, so applying a quasisteady-state argument, we can approximate *Q* using the equation:

(A.1) $$Q=\frac{\alpha_{PL}}{\delta_{Q}}P_{D}L_{\text{free}}=\frac{\alpha_{PL}}{\delta_{Q}}P_{D}\left(L-L_{\text{bound}}\right).$$

We also considered the following reaction:

$$L_{\text{free}}\underset{\kappa_{-1}}{\overset{\kappa_{+1}}{⇌}}L_{\text{bound}},$$

where *k*_+1_ and *k*_−1_ are the association and dissociation rates of *L*_bound_. By the law of mass action and assuming the process is at equilibrium [20],

(A.2) $$\frac{dL_{\text{bound}}}{dt}=k_{+1}L_{\text{free}}A-k_{-1}L_{\text{bound}}=0\;L_{\text{bound}}=\frac{k_{+1}}{k_{-1}}L_{\text{free}}A=\frac{k_{+1}}{k_{-1}}\left(L-L_{\text{bound}}\right)A,\;L_{\text{bound}}=\frac{A}{A+K_{D}}L$$

where $K_{D}=\frac{k_{-1}}{k_{+1}}$ (i.e., the dissociation constant of the PD-L1/anti-PD-L1 complex). Thus, by substituting Equation (A-2) into Equation (A-1), we derived the following expression for *Q*, given by

$$Q=\frac{\alpha_{PL}}{\delta_{Q}}P_{D}L\left(1-\frac{A}{A+K_{D}}\right).$$

Finally, we choose the following functional form for *F*(*Q*) defined by

(A.3) $$F(Q)=\frac{1}{1+\frac{Q}{K_{TQ}}}\equiv F\left(P_{D},L,A\right)\;=\frac{1}{1+\frac{P_{D}L}{K_{YQ}}\left(1-\frac{A}{A+K_{D}}\right)},$$

where $K_{YQ}=\frac{\delta_{Q}}{\alpha_{PL}}K_{TQ}$ (described in Table 2) [18, 20, 21, 24]. In order to achieve agreement between the units for *A* and *K*_*D*_ in Equation (A-3), we converted the dosage of anti-PD-L1 in the experiment from μg to nmol/L using the following formula:

$$c\;(\text{nmol}/\text{L})=\frac{m(\mu \text{g})}{V(L)\times \text{molar}\;\text{mass}(\mu \text{g}/\text{nmol})},$$

where *V* is the carrying capacity of tumor volume without FGFR3 mutation (4000 mm^3^ = 0.004 L), molar mass = 1.5 × 10^5^ g/mol = 1.5 × 10^2^ μg/nmol, so that 100 μg of anti-PD-L1 is equivalent to 166.67 nmol/L of anti-PD-L1.

## APPENDIX B

### B.1 | Pharmacokinetics of anti-FGFR3 (rogaratinib)

We developed a compartmental model to describe the pharmacokinetic profile of rogaratinib in the plasma. The pharmacokinetic model is given as follows, where *G*(*t*), *C*_*S*_(*t*), and *C*_*P*_(*t*) represent the concentration of the drug in the gut, central, and peripheral compartments, respectively:

(B.1) $$\frac{dG}{dt}=k_{a}G\;\frac{dC_{S}}{dt}=Fk_{a}G-k_{12}C_{S}+k_{21}C_{P}-kC_{S}\;\frac{dC_{P}}{dt}=k_{12}C_{S}-k_{21}C_{P},$$

where *k*_*a*_ is the first-order absorption rate constant, *k* is the elimination rate constant, *F* is the bioavailability of the drug that accounts for the fraction of dose that reaches the central compartment, and *k*_12_ and *k*_21_ are distribution rate constants from the central compartment to the peripheral compartment and vice versa, respectively. The pharmacokinetic parameters are estimated by fitting the analytical solution of the central compartment, (*C_S_*(*t*)) to the experimental data of the oral administration of rogaratinib described in [25]. The best fit values and fitting are given in Table B-1 and Figure B-1, respectively.

**TABLE B. 1:** Best fit pharmacokinetic parameter of rogaratinib for PK model (B-1)

Parameter	Description	Best fit values			Reference
		75 mg/kg	50mg/kg	25 mg/kg	
*k*_*a*_	Absorption rate	0.4815 h^−1^	0.3597 h^−1^	0.4942 h^−1^	Best fit
*F*	Bioavailabilty	0.42 h^−1^	0.42 h^−1^	0.42 h^−1^	[27]
*k*_12_	Plasma-tissue transfer rate	577.44 h^−1^	423.11 h^−1^	47.915 h^−1^	Best fit
*k*_21_	Tissue-plasma transfer rate	1.2478 h^−1^	2.6785 h^−1^	0.2864 h^−1^	Best fit
*k*	Elimination rate	193.53 h^−1^	202.95 h^−1^	309.15 h^−1^	Best fit

## APPENDIX C

### C.1 | Model equations related to treatment with anti-FGFR3 (rogaratinib)

The system of equations governing the dynamics of the FGFR3 monomers and dimers (see Figure C-1) in the tumor cell in the presence of rogaratinib is given by

(C.1) $$\frac{dR_{F}}{dt}=-2k_{f}R_{F}^{2}+2k_{r}D_{A}+2k_{p}\left(D_{A}+D_{C}^{C}+D_{A}^{C}\right)+R_{T}P\left(T,\phi_{D}\right)-k_{c,on}^{R}CR_{F}+k_{c,off}^{R}R_{F}^{C}-\frac{R_{F}}{\Sigma }R_{T}D\left(T,Y,\phi_{D}^{C}\right),\;\frac{dD_{A}}{dt}=k_{f}R_{F}^{2}-k_{r}D_{A}-k_{p}D_{A}-k_{c,on}^{D}CD_{A}+k_{c,off}^{D}D_{A}^{C}-\frac{D_{A}}{\Sigma }R_{T}D\left(T,Y,\phi_{D}^{C}\right),\;\frac{dC}{dt}=k_{12}C_{1}-k_{21}C-k_{c,on}^{R}CR_{F}+k_{c,off}^{R}R_{F}^{C}-k_{c,on}^{D}CD_{A}+k_{c,off}^{D}D_{A}^{C},\;\frac{dR_{F}^{C}}{dt}=k_{c,on}^{R}CR_{F}-k_{c,off}^{R}R_{F}^{C}-2k_{f}{\left(R_{F}^{C}\right)}^{2}+2k_{r}D_{C}^{C}-\frac{R_{F}^{C})}{\Sigma }R_{T}D\left(T,Y,\phi_{D}^{C}\right),\;\frac{dD_{C}^{C}}{dt}=k_{f}{\left(R_{F}^{C}\right)}^{2}-k_{r}D_{C}^{C}-k_{p}D_{C}^{C}-\frac{D_{C}^{C}}{\Sigma }R_{T}D\left(T,Y,\phi_{D}^{C},\right)\;\frac{dD_{A}^{C}}{dt}=k_{c,on}^{D}CD_{A}-k_{c,off}^{D}D_{A}^{C}-k_{p}D_{A}^{C}-\frac{D_{A}^{C}}{\Sigma }R_{T}D\left(T,Y,\phi_{D}^{C}\right),$$

where *R*_*F*_, *D*_*A*_, $R_{F}^{C}$, and $D_{A}^{C}$ represent the free FGFR3 monomers, active dimers, monomer/rogaratinib complex, and active dimer/rogaratinib complex, respectively (see Figure 2 for the flowchart of the mechanism of action of rogaratinib). The monomer/rogaratinib complexes dimerize to form $D_{C}^{C}$, and *C* represents the concentration of rogaratinib in the tumor microenvironment. As an anti-FGFR3 drug, we assumed that rogaratinib binds to the kinase region of FGFR3 monomers (*R*_*F*_) and active dimers (*D*_*A*_) on tumor cells at rates $k_{c,on}^{R}$ (to form a monomer/rogaratinib complex $\left(R_{F}^{C}\right)$) and $k_{c,on}^{D}$ (to form active dimer/rogaratinib complex $\left(D_{A}^{C}\right)$). These complexes dissociate at rates $k_{c,off}^{R}$ and $k_{c,off}^{D}$, respectively. Furthermore, we assume that rogaratinib drug does not affect dimerization, dissociation, and internalization; thus, the monomer/rogaratinib complex $\left(R_{F}^{C}\right)$ dimerizes at a rate *k*_*f*_ to form $D_{C}^{C}$ which can either dissociate at a rate *k*_*r*_, internalized at a rate *k*_*p*_. We assumed that upon internalization, both monomer/rogaratinib and active dimer/rogaratinib complexes are recycled at a rate *k*_*p*_, leaving behind the drug, to reproduce FGFR3 monomers (*R*_*F*_). The term $\phi_{D}^{C}$ is the fractional occupancy of active dimer on a tumor cell with anti-FGFR3 and $\Sigma =R_{F}+2D_{A}+R_{F}^{C}+2D_{C}^{C}+2D_{A}^{C}$. The flowchart and parameter values for model (C-1) are given in Table C-1 and Figure C-1, respectively.

**TABLE C. 1:** Parameter values for anti-FGFR3 therapy

Parameter	Description	Value	Units	Reference
$k_{c,on}^{R}$	Rogaratinib-FGFR3 monomer association rate	1.28 × 10^5^	nmol^−1^ d^−1^	[25]
$k_{c,off}^{R}$	Rogaratinib-FGFR3 monomer dissociation rate	95.04	d^−1^	[25]
$k_{c,on}^{D}$	Rogaratinib-FGFR3 dimer association rate	1.28 × 10^5^	nmol^−1^ d^−1^	[25]
$k_{c,off}^{D}$	Rogaratinib-FGFR3 dimer dissociation rate	95.04	d^−1^	[25]

The underlying assumptions for this equation are (i) the tumor resides in a pharmacokinetic compartment of its own; (ii) the binding rates are the same, independent of cell type; (iii) rogaratinib is transferred into the tumor from the systemic circulation at the same rate as the peripheral tissue, *k*_12_; and (iv) the tumor volume is negligible compared to the volume of a mouse; therefore, the amount of the drug leaking into the bloodstream (at the rate *k*_21_) will not affect the concentration of free rogaratinib in the systemic circulation. Furthermore, the formulation of the model (C-1) assumes that the total number (converted to nmol using molecular weight) of receptors per tumor cell *R*_*T*_ remains constant. Thus, we can ensure that the model equations do conserve FGFR3 by considering the sum:

$$\frac{dR_{F}}{dt}+2\frac{dD_{A}}{dt}+\frac{dR_{F}^{C}}{dt}+2\frac{dD_{C}^{C}}{dt}+2\frac{dD_{A}^{C}}{dt}=\frac{d\Sigma }{dt}=R_{T}\left(P\left(T,\phi_{D}^{C}\right)-D\left(T,Y,\phi_{D}^{C}\right)\right)=R_{T}\frac{dT}{dt}.$$

Therefore, upon integration, we have Σ = *R*_*T*_ × *T*.

## References

[1] Siegel R, Miller K, and Jemal A, Cancer statistics, 2020, CA Cancer J. Clin 70 (2020), no. 1, 7–30. 31912902 10.3322/caac.21590

[2] Raghavan D, Chemotherapy for invasive bladder cancer: Five simple rules learned over 30 years, Bladder Cancer 1 (2015), no. 1, 3–13. 30561439 10.3233/BLC-150010 PMC6218183

[3] Chang J, Lara P, and Pan CX, Progress in personalizing chemotherapy for bladder cancer, Adv. Urol 2012 (2012), 364919 10.1155/2012/364919. 22400017 PMC3287014

[4] Casadei C , Targeted therapies for advanced bladder cancer: New strategies with FGFR inhibitors, Ther. Adv. Med. Oncol 11 (2019), 10.1177/1758835919890285. PMC6878604 31803255

[5] Sheepbouwer C , A multimodal imaging approach for longitudinal evaluation of bladder tumor development in an orthotopic murine model, PLoS ONE 11 (2016), no. 8, e0161284. 27533303 10.1371/journal.pone.0161284 PMC4988778

[6] Qin W , The diverse function of PD-1/PD-l pathway beyond cancer, Front. Immunol 10 (2019), 2298. 10.3389/fimmu.2019.02298. 31636634 PMC6787287

[7] Hsu F, Su C, and Huang K, A comprehensive review of US FDA- approved immune checkpoint inhibitors in urothelial carcinoma, J. Immunol. Res 2017 (2017), 6940546 10.1155/2017/6940546. 29376081 PMC5742464

[8] Kacew A and Sweis RF, Fgfr3 alterations in the era of immunotherapy for urothelial bladder cancer, Front. Immunol 11 (2020), 575258. 10.3389/fimmu.2020.575258. 33224141 PMC7674585

[9] Sun X , Multi-scale agent-based brain cancer modeling and prediction of TKI treatment response: Incorporating EGFR signaling pathway and angiogenesis, BMC Bioinfo. 13 (2012), 218. 10.1186/1471-2105-13-218. PMC3487967 22935054

[10] Ji Z , Predicting the impact of combined therapies on myeloma cell growth using a hybrid multi-scale agent-based model, Oncotarget 8 (2017), no. 5, 7647–7665. 10.18632/oncotarget.13831. 28032590 PMC5352350

[11] Zhang J , Integrating evolutionary dynamics into treatment of metastatic castrate-resistant prostate cancer, Nature Commun. 28 (2017), no. 1, 1816. 10.1038/s41467-017-01968-5. PMC5703947 29180633

[12] Jain H and Nör TLJ, Modeling the VEGF-BCL-2-CXCL8 pathway in intratumoral angiogenesis, Bull. Math. Biol 10 (2009), no. 1, 89–117. 10.1007/s11538-007-9242-9. 17701379

[13] Jain H and Nör TLJ, Quantification of endothelial cell-targeted anti BCL-2 therapy and its suppression of tumor growth and vascularization, Molec. Cancer Therapy 8 (2009), no. 10, 2926–2936. 10.1158/1535-7163.MCT-08-1223. PMC2776748 19808978

[14] Swiecicki P , A phase ii trial of the bcl-2 homolog domain 3 mimetic AT-101 in combination with docetaxel for recurrent, locally advanced, or metastatic head and neck cancer, Invest. New Drugs 34 (2016), no. 4, 481–489. 10.1007/s10637-016-0364-5. 27225873 PMC5036856

[15] Jain HV and Jackson TL, Mathematical modeling of cellular cross-talk between endothelial and tumor cells highlights counter-intuitive effects of VEGF-targeted therapies, Bull. Math. Biol 80 (2018), no. 5, 971–1016. 10.1007/s11538-017-0273-6. 28439752

[16] Imai A , Metronomic dosing of BH3 mimetic small molecule yields robust antiangiogenic and antitumor effects, Cancer Res. 72 (2012), no. 3, 716–725. 10.1158/0008-5472.CAN-10-2873. 22158856 PMC3748951

[17] Nazari F , In silico models accurately predict in vivo response for IL-6 blockade in head and neck cancer, Cancer Res. 80 (2020), no. 7, 1451–1460.fphys.2020.00151. 32041834 10.1158/0008-5472.CAN-19-1846 PMC7127935

[18] Storey KM, Lawler SE, Jackson TL, Modeling oncolytic viral therapy, immune checkpoint inhibition, and the complex dynamics of innate and adaptive immunity in glioblastoma treatment, Front. Physiol 11 (2020), 151. 10.3389/fphys.2020.00151. 32194436 PMC7063118

[19] Kuznetsov V , Nonlinear dynamics of immunogenic tumors: Parameter estimation and global bifurcation analysis, Bull. Math. Biol 52 (1994), no. 2, 295–321. 10.1007/BF02460644 8186756

[20] Nikolopoulou E , Mathematical modeling of an immune checkpoint inhibitor and its synergy with an immunostimulant, DCSB-B 22 (2017), no. 11. 10.3934/dcdsb.2020138.

[21] Lai X and Friedman A, Combination therapy of cancer with cancer vaccine and immune checkpoint inhibitors: A mathematical model, PLoS ONE 12 (2017), no. 5, e0178479. 28542574 10.1371/journal.pone.0178479 PMC5444846

[22] Zhao B , Endothelial cell capture of heparin-binding growth factors under flow, PLoS Comput. Biol 6 (2010), no. 10, e1000971. 21060855 10.1371/journal.pcbi.1000971 PMC2965741

[23] Olwin B and Hauschka S, Cell surface fibroblast growth factor and epidermal growth factor receptors are permanently lost during skeletal muscle terminal differentiation in culture, J. Cell. Biol 107 (1988), 761–769. 2843547 10.1083/jcb.107.2.761 PMC2115215

[24] Nikolopoulou E , Tumour-immune dynamics with an immune checkpoint inhibitor, Lett. Biomathem 5 (2018), supll, S137–S159. 10.1080/23737867.2018.1440978.

[25] Grünewald S , Rogaratinib: A potent and selective pan-FGFR inhibitor with broad antitumor activity in FGFR-overexpressing preclinical cancer models, Int. J. Cancer 45 (2019), no. 5, 1346–1357. 10.1002/ijc.32224 PMC6766871 30807645

[26] Schuler M , Rogaratinib in patients with advanced cancers selected by FGFR mRNA expression: A phase 1 dose-escalation and dose-expansion study, The Lancet Oncology 20 (2019), no. 10, 1454–1466. 10.1016/S1470-2045(19)30412-7. 31405822

[27] Collin MP , Discovery of rogaratinib (bay 1163877): A pan-FGFR inhibitor, ChemMedChem 13 (2018), no. 5, 437–445. 29451369 10.1002/cmdc.201700718

[28] Grasselly C , The antitumor activity of combinations of cytotoxic chemotherapy and immune checkpoint inhibitors is model-dependent, Front. Immunol 9 (2018), no. 9, 2100. 10.3389/fimmu.2018.02100. 30356816 PMC6190749

[29] Twyman-Saint V , Radiation and dual checkpoint blockade activate non-redundant immune mechanisms in cancer, Nature 520 (2015), no. 7547, 373–377. 10.1038/nature14292. 25754329 PMC4401634

[30] Javle M , Phase ii study of bgj398 in patients with fgfr-altered advanced cholangiocarcinoma, J Clin Oncol 36 (2018), no. 3, 276–282. 10.1200/JCO.2017.75.5009. 29182496 PMC6075847

[31] Cariboni J , The role of sensitivity analysis in ecological modelling, Ecol. Modell 203 (2007), no. 1–2, 167–182.

[32] Blower SM and Dowlatabadi H, Sensitivity and uncertainty analysis of complex models of disease transmission: An HIV model, as an example, Int. Stat. Rev 62 (1994), no. 2, 229–243.

[33] Marino S , A methodology for performing global uncertainty and sensitivity analysis in systems biology, J. Theor. Biol 254 (2008), no. 1, 178–196. 18572196 10.1016/j.jtbi.2008.04.011 PMC2570191

[34] Eisenburg M and Jain H, A confidence building exercise in data and identifiability: Modeling cancer chemotherapy as a case study, J. Theor. Biol 431 (2017), 63–78. 28733187 10.1016/j.jtbi.2017.07.018 PMC6007023

[35] Gabor A, Villaverde A, and Banga J, Parameter identifiability analysis and visualization in large-scale kinetic models of biosystems, BMC Syst. Biol 11 (2017), 10.1186/s12918-017-0428-y. PMC5420165 28476119

[36] Gallaher J , Methods for determining key components in a mathematical model for tumor–immune dynamics in multiple myeloma, J. Theor. Biol 458 (2018), 31–46. 10.1016/j.jtbi.2018.08.037. 30172689

[37] Meshkat N, Er-z. Kuo C, and DiStefano J III, On finding and using identifiable parameter combinations in nonlinear dynamic systems biology models and combos: A novel web implementation, PLoS ONE 9 (2014), no. 10, e110261. 25350289 10.1371/journal.pone.0110261 PMC4211654

[38] Chis OT, Banga J, and Balsa-Canto E, Structural identifiability of systems biology models: A critical comparison of methods, PLoS ONE 6 (2011), no. 11, e27755. 22132135 10.1371/journal.pone.0027755 PMC3222653

[39] Raue A , Structural and practical identifiability analysis of partially observed dynamical models by exploiting the profile likelihood, Bioinformatics 25 (2009), no. 25, 1923–1929. 19505944 10.1093/bioinformatics/btp358

[40] Chiş O, Banga JR, and Balsa-Canto E, Genssi: A software toolbox for structural identifiability analysis of biological models, Bioinformatics 27 (2011), no. 18, 2610–2611. 21784792 10.1093/bioinformatics/btr431 PMC3167050

[41] de Pillis LG, Radunskaya AE, and Wiseman CL, A validated mathematical model of cell-mediated immune response to tumor growth, Cancer Res. 65 (2005), no. 17, 7950–7958. 16140967 10.1158/0008-5472.CAN-05-0564

[42] Mehrara E , Specific growth rate versus doubling time for quantitative characterization of tumor growth rate, Cancer Res. 67 (2007), no. 8, 3970–3975. 17440113 10.1158/0008-5472.CAN-06-3822

[43] Talkington A and Durrett R, Estimating tumor growth rates in vivo, Bull. Math. Biol 77 (2015), no. 10, 1934–1954. 26481497 10.1007/s11538-015-0110-8 PMC4764475

[44] Tan S , Distinct PD-l1 binding characteristics of therapeutic monoclonal antibody durvalumab, Protein Cell 9 (2018), no. 1, 135–139. 28488247 10.1007/s13238-017-0412-8 PMC5777972

[45] Rosenberg J , Safety and preliminary efficacy of rogaratinib in combination with atezolizumab in a phase Ib/II study (FORT-2) of first-line treatment in cisplatin-ineligible patients (pts) with locally advanced or metastatic urothelial cancer (UC) and FGFR mRNA overexpression, J. Clin. Oncol 38 (2020), 15_suppl, 5014–5014. 10.1200/JCO.2020.38.15_suppl.5014.

[46] Goel M, Khanna P, and Kishore J, Understanding survival analysis: Kaplan-Meier estimate, Int. J. Ayurveda Res 1 (2010), no. 4, 274–278. 21455458 10.4103/0974-7788.76794 PMC3059453

[47] Bellmunt J , ESMO Guidelines Working Group. Bladder cancer: ESMO practice guidelines for diagnosis, treatment and follow-up, Ann. Oncol Suppl 3 (2014), iii40–iii48. 10.1093/annonc/mdu223. 25096609

[48] Flaig TW , Bladder cancer, version 3.2020, NCCN clinical practice guidelines in oncology, J. Natl. Compr. Canc. Netw 3 (2020), 329–354. 10.6004/jnccn.2020.0011. 32135513

[49] Dovedi S and Davies B, Emerging targeted therapies for bladder cancer: A disease waiting for a drug, Cancer Metastasis Rev. 28 (2009), no. 3–4, 355–367. 19997963 10.1007/s10555-009-9192-9

[50] Stenehjem D , PD1/PDL1 inhibitors for the treatment of advanced urothelial bladder cancer, Oncol. Targets Ther 11 (2018), 5973–5989. 10.2147/OTT.S135157 PMC6157986 30275703

[51] Fassan M , Targeted therapies in the management of metastatic bladder cancer, Biologics 1 (2007), no. 4, 333–406. PMC2721287 19707309

